# Exploring Regulatory Mechanisms of Atrial Myocyte Hypertrophy of Mitral Regurgitation through Gene Expression Profiling Analysis: Role of NFAT in Cardiac Hypertrophy

**DOI:** 10.1371/journal.pone.0166791

**Published:** 2016-12-01

**Authors:** Tzu-Hao Chang, Mien-Cheng Chen, Jen-Ping Chang, Hsien-Da Huang, Wan-Chun Ho, Yu-Sheng Lin, Kuo-Li Pan, Yao-Kuang Huang, Wen-Hao Liu, Chia-Chen Wu

**Affiliations:** 1 Graduate Institute of Biomedical Informatics, Taipei Medical University, Taipei, Taiwan; 2 Division of Cardiology and Department of Internal Medicine, Kaohsiung Chang Gung Memorial Hospital, Chang Gung University College of Medicine, Kaohsiung, Taiwan; 3 Division of Cardiovascular Surgery, Kaohsiung Chang Gung Memorial Hospital, Chang Gung University College of Medicine, Kaohsiung, Taiwan; 4 Institute of Bioinformatics and Systems Biology, National Chiao Tung University, Hsinchu, Taiwan; 5 Division of Cardiology, Chang Gung Memorial Hospital, Chiayi, Taiwan; 6 Department of Thoracic and Cardiovascular Surgery, Chang Gung Memorial Hospital, Chiayi, Taiwan; Scuola Superiore Sant'Anna, ITALY

## Abstract

**Background:**

Left atrial enlargement in mitral regurgitation (MR) predicts a poor prognosis. The regulatory mechanisms of atrial myocyte hypertrophy of MR patients remain unknown.

**Methods and Results:**

This study comprised 14 patients with MR, 7 patients with aortic valve disease (AVD), and 6 purchased samples from normal subjects (NC). We used microarrays, enrichment analysis and quantitative RT-PCR to study the gene expression profiles in the left atria. Microarray results showed that 112 genes were differentially up-regulated and 132 genes were differentially down-regulated in the left atria between MR patients and NC. Enrichment analysis of differentially expressed genes demonstrated that “NFAT in cardiac hypertrophy” pathway was not only one of the significant associated canonical pathways, but also the only one predicted with a non-zero score of 1.34 (i.e. activated) through Ingenuity Pathway Analysis molecule activity predictor. Ingenuity Pathway Analysis Global Molecular Network analysis exhibited that the highest score network also showed high association with cardiac related pathways and functions. Therefore, 5 NFAT associated genes (PPP3R1, PPP3CB, CAMK1, MEF2C, PLCE1) were studies for validation. The mRNA expressions of PPP3CB and MEF2C were significantly up-regulated, and CAMK1 and PPP3R1 were significantly down-regulated in MR patients compared to NC. Moreover, MR patients had significantly increased mRNA levels of PPP3CB, MEF2C and PLCE1 compared to AVD patients. The atrial myocyte size of MR patients significantly exceeded that of the AVD patients and NC.

**Conclusions:**

Differentially expressed genes in the “NFAT in cardiac hypertrophy” pathway may play a critical role in the atrial myocyte hypertrophy of MR patients.

## Introduction

Mitral regurgitation (MR) is an important cause of heart failure related to valvular heart disease [1]. Left atrial enlargement has prognostic significance in MR patients undergoing mitral valve surgery [2]. Structural remodeling associated with atrial enlargement, especially pathological hypertrophy of myocytes, developed in the left atrial myocardium of patients with MR [3,4]. However, the molecular regulatory mechanisms and functional biological pathways related to the left atrial myocyte hypertrophy of MR patients remain unclear.

In this study, we aimed to systemically explore the crucial differences in the RNA expression pattern between the left atrial myocardium of MR patients and normal subjects, and the molecular regulatory mechanisms and functional biological pathways related to the atrial myocyte hypertrophy using high-density oligonucleotide microarrays and enrichment analysis. The left atrial myocardium of patients with severe aortic valve disease was also used as a reference for gene analysis of the significant pathways as the left atrial size was smaller in patients with aortic valve disease compared to MR patients. The results of this study may recognize some of the differentially expressed genes and related pathways that contribute to the left atrial myocyte hypertrophy in patients with MR.

## Methods

### Patient Population

This study enrolled 14 patients with symptomatic severe non-ischemic MR in sinus rhythm (age: 58±9 years), and 7 age-matched patients with symptomatic severe aortic valve disease in sinus rhythm (age: 63±7 years; aortic stenosis in 1, aortic regurgitation in 4, combined aortic stenoregurgitation in 2). Exclusion factors include previous myocardial infarction, febrile disorder, infectious or inflammatory disease, autoimmune disease, malignancy, acute or chronic viral hepatitis or use of immunosuppressive drugs. Written informed consent was obtained from each study patient, and the study protocol conforms to the ethical guidelines of the 1975 Declaration of Helsinki as reflected in a priori approval by the Institutional Review Board of Chang Gung Memorial Hospital (100-0067C).

Six normal adult left atrial tissue samples (24-year-old Caucasian male, 27-year-old Caucasian male, 30-year-old Asian male, 60-year-old Caucasian female, 76-year-old Caucasian female and 77-year-old Caucasian male) were purchased from BioChain, Newark, CA, USA, and these 6 normal atrial tissues were used as the normal controls for gene analysis.

### Specimen Storage

Atrial tissues of non-ischemic MR patients and aortic valve disease patients with heart failure were sampled from the left atrial free wall during surgery. After excision, some atrial tissues were immediately frozen in liquid nitrogen and stored at –80 Celsius, and some were immediately fixed in 3.7% buffered formalin, then embedded in paraffin, and stored until later study for hematoxylin/eosin staining.

### Microarray Analysis and Data Processing

RNAs were extracted from the myocardial samples by using a RiboPureTM kit (Ambion, Grand Island, NY, USA) according to the manufacturer's protocol. RNA quality was assessed using an Agilent 2100 Bioanalyzer (Agilent Technologies Inc, Santa Clara, CA, USA). Samples with optical density ratio 260/280 > 1.8 and RNA integrity number > 7.0 were selected and sent for microarray processing. Two hundred fifty ng of total RNA per sample was used for cRNA production by the RiboPure^TM^RNA extraction kit (Ambion, Grand Island, NY, USA). The quality of cRNA was evaluated using the RNA 6000 pico kit (Agilent Technologies, Santa clara,CA,USA) and the Experion automated electrophoresis station (Bio-Rad Laboratories, Inc., Hercules, CA, USA). A total of 750 ng cRNA was used for hybridization to a human HT12-v4 Illumina Beadchip gene expression array (Illumina, San Diego, CA, USA), including 47231 probes and 28688 annotated genes, according to the manufacturer’s protocol. The arrays were scanned and fluorescence signals obtained using the Illumina Bead Array Reader (Illumina, San Diego, CA, USA). Microarray quality control and normalization was performed using Illumina GenomeStudio data analysis software. The expression level of a gene was represented by the average probe intensity. Functional classes were assigned to all known genes using information from the Gene Ontology database available at the website (http://amigo.geneontology.org/cgi-bin/amigo/go.cgi). Additionally, we applied the activation z-score analysis method [5] to measure activation states (increased or decreased) of the pathways affected by differentially expressed genes. The sign of the calculated z-score will reflect the overall predicted activation state of the biological function (<0: decreased, >0: increased).

### Quantitative Determination of RNAs by Real-Time RT-PCR

The RNA samples were quantified using a spectrophotometer. First-strand cDNAs were synthesized with reverse transcriptase and oligo (dT) primers. Real-time quantitative PCR was performed on the ABI Prism 7500 FAST sequence detection system (Applied Biosystems, CA, USA), using SYBR Green PCR Master Mix (Applied Biosystems, CA, USA). The results of RNAs were normalized against 18S gene expression (the endogenous control). The selected genes and primer sequences are presented in Table 1. The microRNAs (miRs) were extracted from the tissues by using a RNA MiniPrep kit (Zymo Research, CA, USA) according to the manufacturer’s protocol. Reverse transcription of miRs was performed using the TaqMan™ microRNA reverse transcription kit (Applied Biosystems, CA, USA) according to manufacturer's recommendations. Briefly, 5 ng of miR was combined with deoxyribonucleoside triphosphates, MultiScribe™ reverse transcriptase, and the primer specific for the target miR (Applied Biosystems, CA, USA). The cDNA was combined with the TaqMan™ assay specific for the target miR. The results of miRs were normalized against U6 snRNA (Applied Biosystems, CA, USA). Quantitative RT-PCR values were presented in △Cq units.

**Table 1:** Primer Sequences for Real-Time PCR.

Gene Name	Forward Primer	Reverse Primer
Human		
PPP3CB	TGGTGGACTTTCACCAGAAAT	GCAGGTGGCTCTTTGAATCT
PLCE1	CTGCGGAAACAGTACGTCAG	CAAAGTTGGGCCTTCATACC
CAMK1	AAGGCAGCATGGAGAATGAG	GCTACAATGTTGGGGTGCTT
PPP3R1	TTATAATCCCAGCCAGTGGTTT	AAAGGCTAGTTCCCCCTTGA
MEF2C	AAGGTATCCATGGAACATGAAAG	TGAGTGTGTATATTTTCAGGGATGTT
18S	GTAACCCGTTGAACCCCATT	CCATCCAATCGGTAGTAGCG
Mouse		
CAMK1	AAGCAGGCGGAAGACATTAG	TCCTCTGCCAGGATCACTTC
PPP3R1	TGTAGACAAAACCATAATAAATGCAGA	GGATATCTAGGCCACCTACGAC
GAPDH	AGCTTGTCATCAACGGGAAG	TTTGATGTTAGTGGGGTCTCG

CAMK1 = calcium/calmodulin-dependent protein kinase I; PLCE1 = phospholipase C, epsilon 1; PPP3CB (Calcineurin A beta) = protein phosphatase 3, catalytic subunit, beta isozyme; PPP3R1 (Calcineurin B) = protein phosphatase 3, regulatory subunit B, alpha; MEF2C = myocyte enhancer factor 2C.

### Western Blotting

The protein extracts of human atrial tissues were examined by Western blot analysis. 20μg protein extracts were electrophoresed on 10% acrylamide SDS-PAGE gels and immunoblotted onto polyvinylidene difluoride membranes. The membranes were preblocked for 1 h in TBST (10 mM Tris–HCl pH 7.6, 150 mM NaCl, 0.1% Tween-20) containing 5% w/v nonfat dry milk and then incubated at 4°C overnight with anti-α-sarcomeric actin (Sigma Aldrich, Louis, MO, USA). The result of protein was normalized against GAPDH.

### Histological Analysis

Atrial tissue sections were deparaffinized in xylene and rehydrated in decreasing concentrations of alcohol. Slides were then stained with hematoxylin/eosin. Tissue sections were observed under an Olympus BX51 microscope with the analysis including at least 100 randomly selected cells under 400 X magnification. All images of each specimen were captured using an Olympus DP70 camera. Atrial cardiomyocytes were analyzed (UTHSCSA, Image tool, Version 3.0).

### Cell Culture and Mechanical Stretching of HL-1 Atrial Myocytes

HL-1 atrial myocytes were plated on silicone rubber culture dishes. HL-1 atrial myocytes were cultured for 24 hours in claycomb medium containing 10% FBS, penicillin and streptomycin. Thereafter, culture medium was changed to serum free claycomb medium and HL-1 atrial myocytes were stretched for an additional 8 hours in the same medium. HL-1 atrial myocytes received 15% uniaxial cyclic stretch at 1 Hz for 8 hours by NST-140 cell stretching system (NEPA GENE, Japan) in the cell stretched study groups. Stretched and control (non-stretched) experiments were carried out simultaneously with the same pool of cells in each experiment to match temperature, CO_2_ content, and pH of the medium for the stretched and control HL-1 atrial myocytes.

### Immunofluorescence Staining

HL-1 atrial myocytes were fixed for 10 min with 4% paraformaldehyde, then exposed to 0.1% triton-X100, and stained with CytoPainter Phalloidin-iFluor 488 reagent (Abcam, Cambridge, USA), according to the manufacturer's protocol. Nuclei were stained with Hoechst 33258 (1:1000 dilution; Sigma, MO, USA). Four randomly chosen fields per section corresponding to at least fifty cells were examined at high magnification (400X). All images of each specimen were captured using a Leica DMI3000 microscope. Atrial cardiomyocytes were analyzed (UTHSCSA, Image tool, Version 3.0).

### Statistical Analysis

Data are presented as mean ± SD (baseline characteristics) or SEM (gene and protein expressions). Categorical variables were compared using chi-square test or Fisher exact test as appropriate. Continuous variables among 3 groups were analyzed by the Kruskal-Wallis Test, and continuous variables between 2 groups were analyzed by the Mann-Whitney Test. Statistical analysis was performed using commercial statistical software (IBM SPSS Statistics 22). All *P* values were two-sided, and the level of statistical significance was set at 0.05.

## Results

### Baseline Characteristics of Patients Studied

Table 2 lists the clinical characteristics of the study patients with MR and patients with aortic valve disease. The two groups did not significantly differ in age, or heart failure status. The two groups also did not significantly differ in the preoperative left atrial ejection fraction, left ventricular size and left ventricular ejection fraction. However, the left atrial size was significantly larger in the MR patients than patients with aortic valve disease. Seventy-eight percent of MR patients and forty-two percent of patients with aortic valve disease received renin-angiotensin system blockers (*P* = 0.102).

**Table 2:** Baseline Clinical Characteristics of the Study Patients.

	MR (n = 14)	AVD (n = 7)	*P* value
Age (years)	58±9	60±11	0.550
Male (%)	6 (42.9%)	6 (85.7%)	0.159
Body mass index (kg/m^2^)	23.6±2.4	24.2±3.3	0.314
Hypertension (%)	7 (50.0%)	4 (57.1%)	1.000
Diabetes mellitus (%)	2 (14.3%)	1 (14.3%)	1.000
Dyslipidemia (%)	6 (42.9%)	2 (28.6%)	0.656
Heart failure NYHA classification			0.522
Functional class I (%)	2 (14.3%)	1 (14.3%)	
Functional class II (%)	6 (42.9%)	3 (42.9%)	
Functional class III (%)	6 (42.9%)	2 (28.6%)	
Functional class IV (%)	0 (0.0%)	1 (14.3%)	
Tricuspid regurgitation (%)	6 (42.9%)	1 (14.3%)	0.337
Beta-blockers (%)	4 (28.6%)	0 (0.0%)	0.255
Calcium channel blockers (%)	6 (42.9%)	3 (42.9%)	1.000
Angiotensin converting enzyme inhibitors or angiotensin II receptor blockers (%)	11 (78.6%)	3 (42.9%)	0.102
Statins (%)	1 (7.1%)	0 (0.0%)	1.000
Creatinine (mg/dl)	0.9±0.7	1.0±0.3	0.101
White blood cell count (10^3^/uL)	6.3±1.6	5.6±1.8	0.331
Left atrial diameter (mm)	45.9±6.0	38.9±5.8	0.020
Left atrial maximal volume (mL)	88.1±44.1	42.5±25.6	0.037
Left atrial ejection fraction (%)	51.4±10.6	45.6±18.7	0.501
Left ventricular end-diastolic diameter (mm)	58.6±7.3	59.9±12.7	0.477
Left ventricular ejection fraction (%)	68.1±11.4	61.6±12.9	0.295

Data are presented as mean ± SD or number (percentage).

AVD = aortic valve disease; MR = mitral regurgitation; NYHA = New York Heart Association.

### Identification and Enrichment Analysis of Differential Expression Genes between MR Patients and Normal Subjects

**Fig 1:**
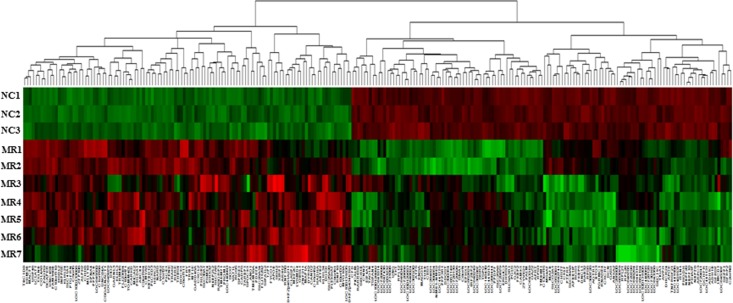
Unsupervised hierarchical clustering of RNA microarray expression values. A total of 112 genes were identified to be differentially up-regulated and 132 genes were identified to be differentially down-regulated in the left atria between mitral regurgitation (MR) patients (n = 7) and normal subjects (NC) (n = 3) by using *genefilter* R package with the *P* value < 0.01 (t-test) and a fold-change cut-offs of > 1.5. Bar color indicates mRNA expression level. Red indicates up-regulation; black, no change; green, down-regulation.

To determine the effect of MR on gene expression, we compared the expression profile in the left atrial free walls of the 7 MR patients to 3 normal subjects (76-year-old Caucasian female, 24-year-old Caucasian male and 27-year-old Caucasian male). A total of 244 differentially expressed genes were discovered by using *genefilter* R package [6] with the *P* value < 0.01 (t-test) and a fold-change cut-offs of > 1.5. A total of 112 genes were identified to be differentially up-regulated between MR patients and normal subjects (Table 3), and a total of 132 genes were identified to be differentially down-regulated between MR patients and normal subjects (Table 4), with the heat map graph being depicted in Fig 1. As with the unsupervised hierarchical clustering, the samples and genes were sorted corresponding to their respective groups.

**Table 3:** Selected Signature Upregulated Gene Expression in the Left Atria of Mitral Regurgitation vs. Normal Control.

Symbol	Entrez ID	Gene Title	Gene Ontology	KEGG Pathway	Log_2_FC(MR/NC)
TMEM71	137835	transmembrane protein 71	CC: membrane		1.879
DKFZp451A211	400169	DKFZp451A211 protein			1.787
XIRP1	65904	xin actin-binding repeat containing 1	BP: negative regulation of cell proliferation, regulation of membrane potential, sarcomere organization, cardiac muscle cell development; CC: cell-cell adherens junction, fascia adherens; MF: actin binding, protein binding, poly(A) RNA binding		1.689
PROM1	8842	prominin 1	CC: extracellular space, integral component of plasma membrane, cell surface, stereocilium, endoplasmic reticulum; MF: actinin binding, cadherin binding		1.587
CXCL2	2920	chemokine (C-X-C motif) ligand 2	BP: immune response, inflammatory response, chemokine activity, positive regulation of leukocyte chemotaxis, chemokine-mediated signaling pathway, G-protein coupled receptor signaling pathway, positive regulation of cytosolic calcium ion concentration, regulation of cell proliferation, cell chemotaxis; CC: extracellular region, cytosol; MF: CXCR chemokine receptor binding, cytokine activity	TNF signaling pathway, Cytokine-cytokine receptor interaction, Chemokine signaling pathway, NF-kappa B signaling pathway, NOD-like receptor signaling pathway	1.377
PLCE1	51196	phospholipase C, epsilon 1	BP: activation of MAPK activity, calcium-mediated signaling, cell proliferation, cytoskeleton organization, diacylglycerol biosynthetic process, heart development, inositol phosphate metabolic process, phospholipase C-activating G-protein coupled receptor signaling pathway, positive regulation of cytosolic calcium ion concentration, protein kinase C-activating G-protein coupled receptor signaling pathway, Ras protein signal transduction, regulation of cell growth, regulation of G-protein coupled receptor protein signaling pathway, regulation of protein kinase activity; CC: cytoplasm, Golgi membrane, plasma membrane; MF: calcium ion binding, guanyl-nucleotide exchange factor activity, phosphatidylinositol phospholipase C activity, phospholipase C activity, protein binding, Ras GTPase binding, receptor signaling protein activity	Phosphatidylinositol signaling system, Ras signaling pathway, Inositol phosphate metabolism, Metabolic pathways, Rap1 signaling pathway, Calcium signaling pathway, cAMP signaling pathway	1.333
PLCXD3	345557	phosphatidylinositol-specific phospholipase C, X domain containing 3	BP: lipid metabolic process, signal transduction; MF: phosphoric diester hydrolase activity, signal transducer activity		1.270
C10orf71	118461	chromosome 10 open reading frame 71			1.200
RGS5	8490	regulator of G-protein signaling 5	BP: positive regulation of GTPase activity, regulation of G-protein coupled receptor protein signaling pathway; CC: cytoplasm, plasma membrane; MF: GTPase activator activity		1.168
C9orf61	9413	chromosome 9 open reading frame 61			1.153
CMYA5	202333	cardiomyopathy associated 5	BP: negative regulation of calcineurin-NFAT signaling cascade, negative regulation of protein phosphatase type 2B activity; CC: costamere, M band, perinuclear region of cytoplasm; MF: protein binding		1.127
PLEKHA7	144100	pleckstrin homology domain containing, family A member 7	BP: epithelial cell-cell adhesion, zonula adherens maintenance; CC: centrosome, cytoplasm, zonula adherens; MF: delta-catenin binding		1.119
GADD45A	1647	growth arrest and DNA-damage-inducible, alpha	BP: G2/M transition of mitotic cell cycle, activation of MAPKKK activity, negative regulation of protein kinase activity, cellular response to DNA damage stimulus, cell cycle arrest, centrosome cycle, signal transduction in response to DNA damage, positive regulation of apoptotic process, positive regulation of JNK cascade, positive regulation of p38MAPK cascade, regulation of cell cycle, positive regulation of reactive oxygen species metabolic process, negative regulation of protein kinase activity, cellular response to mechanical stimulus, DNA repair; CC: nucleus, cytoplasm; MF: core promoter binding, protein binding	FoxO signaling pathway, p53 signaling pathway, MAPK signaling pathway, Cell cycle	1.117
KLHL3	26249	kelch-like 3	BP: protein ubiquitination, protein ubiquitination involved in ubiquitin-dependent protein catabolic process, ion homeostasis, protein K48-linked ubiquitination; CC: cytosol, cytoskeleton, Cul3-RING ubiquitin ligase complex; MF: actin binding, catalytic activity		1.094
DHX32	55760	DEAH (Asp-Glu-Ala-His) box polypeptide 32	BP: mRNA splicing, via spliceosome, RNA processing; CC: cytoplasm, spliceosomal complex, nucleus, mitochondrion, alpha DNA polymerase:primase complex; MF: poly(A) RNA binding, ATP-dependent RNA helicase activity, nucleotide binding, ATP binding		1.076
LOC100133866	100133866				1.074
PHLDB2	90102	pleckstrin homology-like domain, family B, member 2	CC: cytoplasm, plasma membrane, intermediate filament cytoskeleton; MF: protein binding		1.064
HSDL2	84263	hydroxysteroid dehydrogenase like 2	CC: mitochondrion, peroxisome; MF: oxidoreductase activity, reduced coenzyme F420 dehydrogenase activity, NADPH:sulfur oxidoreductase activity, epoxyqueuosine reductase activity. malolactic enzyme activity, N-ethylmaleimide reductase activity		1.056
DIO2	1734	deiodinase, iodothyronine, type II	BP: oxidation-reduction process, thyroid hormone metabolic process, hormone biosynthetic process, response to hormone; CC: integral component of membrane; MF: thyroxine 5'-deiodinase activity, ubiquitin protein ligase binding	Thyroid hormone signaling pathway	1.055
MICAL2	9645	microtubule associated monoxygenase, calponin and LIM domain containing 2	BP: heart looping, cytoskeleton organization, positive regulation of transcription via serum response element binding, actin filament depolymerization, sulfur oxidation; CC: nucleus; MF: actin binding, oxidoreductase activity, acting on paired donors, with incorporation or reduction of molecular oxygen, NAD(P)H as one donor, and incorporation of one atom of oxygen, NADPH:sulfur oxidoreductase activity. FAD binding, monooxygenase activity		1.013
C15orf52	388115	chromosome 15 open reading frame 52	MF: poly(A) RNA binding		1.013
MT1E	4493	metallothionein 1E	BP: negative regulation of growth, cellular response to cadmium ion, cellular response to zinc ion; CC: nucleus, cytoplasm; MF: zinc ion binding, metal ion binding	Mineral absorption	1.008
TBC1D8	11138	TBC1 domain family, member 8	BP: positive regulation of cell proliferation, blood circulation, positive regulation of Rab GTPase activity, regulation of cilium assembly; CC: membrane; MF: calcium ion binding, Rab GTPase activator activity		1.001
CAND2	23066	cullin-associated and neddylation-dissociated 2 (putative)	BP: SCF complex assembly, protein ubiquitination, positive regulation of transcription, DNA-templated; CC: nucleus; MF: protein binding, TBP-class protein binding		0.996
THBS4	7060	thrombospondin 4	BP: positive regulation of endothelial cell proliferation, negative regulation of angiogenesis, regulation of tissue remodeling, response to endoplasmic reticulum stress, positive regulation of peptidyl-tyrosine phosphorylation, endothelial cell-cell adhesion, response to unfolded protein, tissue remodeling, positive regulation of cell division; CC: extracellular region, basement membrane, endoplasmic reticulum, sarcoplasmic reticulum, extracellular matrix, extracellular exosome; MF: integrin binding, calcium ion binding, protein binding, heparin binding, growth factor activity	ECM-receptor interaction, Phagosome, PI3K-Akt signaling pathway, Focal adhesion	0.996
APOB	338	apolipoprotein B	BP: retinoid metabolic process, receptor-mediated endocytosis, cholesterol metabolic process, positive regulation of lipid storage, low-density lipoprotein particle clearance; CC: extracellular region, cytoplasm, early endosome, endoplasmic reticulum lumen, Golgi apparatus, plasma membrane, actin cytoskeleton, clathrin-coated endocytic vesicle membrane; MF: protein binding, phospholipid binding, cholesterol transporter activity, lipase binding	Fat digestion and absorption, Vitamin digestion and absorption	0.991
ZFP106	64397	zinc finger protein 106 homolog	BP: insulin receptor signaling pathway; CC: nucleolus, cytosol, membrane;MF: poly(A) RNA binding, opioid peptide activity, SH3 domain binding, metal ion binding		0.969
RASGRP3	25780	RAS guanyl releasing protein 3 (calcium and DAG-regulated)	BP: Ras protein signal transduction, positive regulation of Ras GTPase activity, regulation of small GTPase mediated signal transduction, MAPK cascade; CC: cytoplasm, guanyl-nucleotide exchange factor complex, perinuclear region of cytoplasm; MF: Ras guanyl-nucleotide exchange factor activity, Ras GTPase binding, Rap GTPase activator activity, calcium ion binding, metal ion binding	Ras signaling pathway, MAPK signaling pathway, Rap1 signaling pathway	0.966
SLC25A34	284723	solute carrier family 25, member 34	BP: transport; CC: mitochondrion		0.964
PHLDA1	22822	pleckstrin homology-like domain, family A, member 1	BP: apoptotic process, FasL biosynthetic process; CC: nucleolus, plasma membrane, cytoplasm; MF: protein binding		0.961
SLC41A1	254428	solute carrier family 41, member 1	BP: cation transport, ion transport, transmembrane transport, CC: plasma membrane; MF: cation transmembrane transporter activity		0.932
FLJ11292	55338				0.912
RNF150	57484	ring finger protein 150	CC: membrane, integral component of membrane; MF: zinc ion binding, metal ion binding		0.907
C6orf111	25957	chromosome 6 open reading frame 111			0.904
UNC84A	23353	unc-84 homolog A	CC: nuclear membrane, intracellular membrane-bounded organelle		0.885
TRIM45	80263	tripartite motif containing 45	CC: nucleus, cytoplasm, intercellular bridge; MF: zinc ion binding, metal ion binding		0.883
VAT1L	57687	vesicle amine transport protein 1 homolog (T. californica)-like	MF: oxidoreductase activity, zinc ion binding		0.880
PTGFRN	5738	prostaglandin F2 receptor negative regulator	BP: lipid particle organization, negative regulation of translation; CC: endoplasmic reticulum, Golgi apparatus, cell surface, membrane; MF: protein binding		0.871
CADPS	8618	Ca^2+^-dependent secretion activator	BP: transport, exocytosis, vesicle organization, synaptic vesicle priming, positive regulation of calcium ion-dependent exocytosis, catecholamine secretion, regulated secretory pathway; CC: membrane, cell junction, cytoplasmic vesicle, synapse; MF: calcium ion binding, protein binding, phosphatidylinositol-4,5-bisphosphate binding, lipid binding, protein kinase binding		0.868
SIPA1L2	57568	signal-induced proliferation-associated 1 like 2	BP: positive regulation of GTPase activity, regulation of small GTPase mediated signal transduction; MF: GTPase activator activity	Rap1 signaling pathway	0.865
KLHL34	257240	kelch-like 34	CC: extracellular space		0.860
ALPK2	115701	alpha-kinase 2	MF: ATP binding, protein serine/threonine kinase activity		0.854
FAM13B	51306	family with sequence similarity 13, member B	BP: positive regulation of GTPase activity, regulation of mitochondrion degradation, small GTPase mediated signal transduction, regulation of small GTPase mediated signal transduction, activation of mitophagy in response to mitochondrial depolarization; CC: cytosol; MF: GTPase activator activity		0.849
SOX7	83595	SRY (sex determining region Y)-box 7	BP: heart development, endoderm formation, negative regulation of cell proliferation, positive regulation of cysteine-type endopeptidase activity involved in apoptotic process, regulation of canonical Wnt signaling pathway, positive regulation of transcription, DNA-templated, negative regulation of transcription, DNA-templated, regulation of transcription from RNA polymerase II promoter; CC: nucleus, cytoplasm; MF: sequence-specific DNA binding transcription factor activity, transcription regulatory region DNA binding, RNA polymerase II distal enhancer sequence-specific DNA binding transcription factor activity		0.845
KLHL24	54800	kelch-like 24	BP: regulation of kainate selective glutamate receptor activity; CC: cytoplasm		0.838
PYROXD2	84795	pyridine nucleotide-disulphide oxidoreductase domain-containing protein 2	BP: oxidation-reduction process; MF: oxidoreductase activity, N-ethylmaleimide reductase activity, reduced coenzyme F420 dehydrogenase activity, sulfur oxygenase reductase activity, malolactic enzyme activity, NADPH:sulfur oxidoreductase activity, epoxyqueuosine reductase activity, N-ethylmaleimide reductase activity		0.821
INPP5E	56623	inositol polyphosphate-5-phosphatase	BP: phospholipid metabolic process, phosphatidylinositol biosynthetic process, phosphatidylinositol dephosphorylation, inositol phosphate dephosphorylation; CC: cytosol, axoneme, Golgi membrane, cytoskeleton; MF: inositol-polyphosphate 5-phosphatase activity, phosphatidylinositol-4,5-bisphosphate 5-phosphatase activity, hydrolase activity	Inositol phosphate metabolism, Metabolic pathways, Phosphatidylinositol signaling system	0.803
TRAK2	66008	trafficking protein, kinesin binding 2	BP: regulation of transcription from RNA polymerase II promoter, protein O-linked glycosylation; CC: cytoplasm, mitochondrion, plasma membrane, nucleus, early endosome; MF: receptor binding, protein binding, GABA receptor binding, enzyme binding	Metabolic pathways, GABAergic synapse	0.803
TRIB1	10221	10221	BP: positive regulation of proteasomal ubiquitin-dependent protein catabolic process, regulation of MAP kinase activity; CC: nucleus; MF: mitogen-activated protein kinase kinase binding		0.800
LOC440993	440993				0.796
SPOCK1	6695	sparc/osteonectin, cwcv and kazal-like domains proteoglycan (testican) 1	BP: negative regulation of cell-substrate adhesion, negative regulation of endopeptidase activity, signal transduction, neurogenesis; CC: extracellular space, cytoplasm, sarcoplasm, neuromuscular junction; MF: cysteine-type endopeptidase inhibitor activity, calcium ion binding, metalloendopeptidase inhibitor activity		0.793
PENK	5179	proenkephalin	BP: neuropeptide signaling pathway, signal transduction; CC: extracellular region; MF: neuropeptide hormone activity, protein binding		0.790
RERE	473	arginine-glutamic acid dipeptide (RE) repeats	BP: NLS-bearing protein import into nucleus, regulation of transcription, DNA-templated, chromatin remodeling; CC: nucleus, histone deacetylase complex; MF: chromatin binding, protein binding, poly-glutamine tract binding, sequence-specific DNA binding transcription factor activity, zinc ion binding		0.769
MEF2C	4208	myocyte enhancer factor 2C	BP: negative regulation of transcription from RNA polymerase II promoter, positive regulation of transcription from RNA polymerase II promoter, MAPK cascade, positive regulation of gene expression, humoral immune response, positive regulation of myoblast differentiation, positive regulation of skeletal muscle tissue development, cellular response to lipopolysaccharide, cellular response to calcium ion, cellular response to transforming growth factor beta stimulus; CC: nucleus, cytoplasm, nuclear speck, protein complex; MF: RNA polymerase II regulatory region sequence-specific DNA binding, RNA polymerase II core promoter sequence-specific DNA binding transcription factor activity, DNA binding, miRNA binding	MAPK signaling pathway, cGMP-PKG signaling pathway	0.767
LOC645979	645979				0.765
MAP1A	4130	microtubule-associated protein 1A	BP: activation of mitophagy in response to mitochondrial depolarization, microtubule cytoskeleton organization; CC: microtubule associated complex, cytosol, microtubule; MF: structural molecule activity, protein binding, microtubule binding		0.760
FRY	10129	furry homolog	CC: spindle pole, cytoplasm, microtubule organizing center		0.758
COL4A6	1288	collagen, type IV, alpha 6	BP: extracellular matrix disassembly, collagen catabolic process, extracellular matrix organization, cellular response to amino acid stimulus; CC: extracellular region, collagen type IV trimer, endoplasmic reticulum lumen, basement membrane; MF: extracellular matrix structural constituent, structural molecule activity	ECM-receptor interaction, PI3K-Akt signaling pathway, Focal adhesion	0.757
LCLAT1	253558	lysocardiolipin acyltransferase 1	BP: metabolic process, phospholipid metabolic process, phosphatidic acid biosynthetic process, triglyceride biosynthetic process, cardiolipin acyl-chain remodeling, glycerophospholipid biosynthetic process, CDP-diacylglycerol biosynthetic process; CC: endoplasmic reticulum, membrane; MF: transferase activity, transferring acyl groups, 1-acylglycerol-3-phosphate O-acyltransferase activity, sterol O-acyltransferase activity	Glycerophospholipid metabolism, Glycerolipid metabolism, Metabolic pathways	0.755
WSB2	55884	WD repeat and SOCS box-containing 2	BP: intracellular signal transduction, protein ubiquitination		0.754
PSME4	23198	proteasome (prosome, macropain) activator subunit 4	BP: anaphase-promoting complex-dependent proteasomal ubiquitin-dependent protein catabolic process, apoptotic process, cellular nitrogen compound metabolic process, cellular response to DNA damage stimulus, DNA damage response, signal transduction by p53 class mediator resulting in cell cycle arrest, DNA repair, gene expression, mRNA metabolic process, multicellular organismal development, positive regulation of peptidase activity, proteasomal ubiquitin-independent protein catabolic process, protein polyubiquitination, regulation of cellular amino acid metabolic process; CC: cytosol, nucleus; MF: histone acetyl-lysine binding, peptidase activator activity	Proteasome	0.752
ABTB2	25841	ankyrin repeat and BTB (POZ) domain containing 2	BP: cellular response to toxic substance; CC: nucleus; MF: protein heterodimerization activity		0.747
SLC7A6	9057	solute carrier family 7 (cationic amino acid transporter, y+ system), member 6	BP: amino acid transport, blood coagulation, cellular amino acid metabolic process, ion transport, protein complex assembly, transmembrane transport; CC: plasma membrane. integral component of membrane; MF: amino acid transmembrane transporter activity, antiporter activity		0.745
TOMM40L	84134	translocase of outer mitochondrial membrane 40 homolog (yeast)-like	BP: ion transport, protein transport, transmembrane transport; CC: mitochondrial outer membrane, pore complex, protein complex; MF: porin activity		0.739
MYO18A	399687	myosin XVIIIA	BP: Golgi organization, cell migration, actomyosin structure organization, negative regulation of apoptotic process, Golgi vesicle budding, positive regulation of protein secretion, DNA metabolic process; CC: Golgi membrane, trans-Golgi network, actomyosin, myosin complex, endoplasmic reticulum-Golgi intermediate compartment; MF: protein binding, ATP binding, poly(A) RNA binding, actin filament binding, motor activity		0.728
LOC100131835	100131835				0.718
C1orf21	81563	chromosome 1 open reading frame 21	MF: protein binding		0.714
KDM3B	51780	lysine (K)-specific demethylase 3B	BP: chromatin modification, transcription, DNA-templated; CC: nucleus; MF: dioxygenase activity, metal ion binding		0.713
CLASP1	23332	cytoplasmic linker associated protein 1	BP: negative regulation of microtubule depolymerization; CC: cytoplasmic microtubule, kinetochore microtubule; MF: kinetochore binding, microtubule plus-end binding		0.708
TMEM16A	55107	transmembrane protein 16A	BP: cation transport, chloride transport, ion transmembrane transport, regulation of membrane potential, phospholipase C-activating G-protein coupled receptor signaling pathway, regulation of anion transmembrane transport; CC: plasma membrane, extracellular vesicular exosome; MF: calcium activated cation channel activity, intracellular calcium activated chloride channel activity, protein binding, protein homodimerization activity, protein heterodimerization activity		0.707
DGKD	8527	diacylglycerol kinase, delta 130kDa	BP: signal transduction, epidermal growth factor receptor signaling pathway, protein kinase C-activating G-protein coupled receptor signaling pathway, cell growth, diacylglycerol metabolic process, protein homooligomerization; CC: cytoplasm, plasma membrane, cytoplasmic membrane-bounded vesicle; MF: diacylglycerol kinase activity, protein binding, diacylglycerol binding, protein heterodimerization activity, protein homodimerization activity, NAD+ kinase activity, diacylglycerol kinase activity, ATP binding	Glycerolipid metabolism, Glycerophospholipid metabolism, Metabolic pathways, Phosphatidylinositol signaling system	0.693
HERC2	8924	HECT and RLD domain containing E3 ubiquitin protein ligase 2	BP: DNA repair, intracellular protein transport, protein ubiquitination, regulation of GTPase activity; CC: cytoplasm, mitochondrial inner membrane, nucleus; MF: guanyl-nucleotide exchange factor activity, heme binding, protein binding, SUMO binding, ubiquitin protein ligase binding, ubiquitin-protein ligase activity	Ubiquitin mediated proteolysis	0.687
DKFZp434K191	29797				0.686
KBTBD12	166348	kelch repeat and BTB (POZ) domain containing 12			0.683
DOCK1	1793	dedicator of cytokinesis 1	BP: cytoskeleton organization, small GTPase mediated signal transduction, cell migration, positive regulation of GTPase activity; MF: guanyl-nucleotide exchange factor activity, GTPase activator activity; CC: intracellular	Focal adhesion, Regulation of actin cytoskeleton	0.681
C1orf168	199920	chromosome 1 open reading frame 168			0.674
RNF10	9921	ring finger protein 10	BP: negative regulation of Schwann cell proliferation, positive regulation of myelination, positive regulation of transcription from RNA polymerase II promoter, positive regulation of transcription, DNA-templated; CC: nucleus, cytoplasm; MF: transcription regulatory region DNA binding, zinc ion binding, protein binding		0.671
CHD6	84181	chromodomain helicase DNA binding protein 6	BP: positive regulation of transcription from RNA polymerase II promoter in response to oxidative stress, metabolic process, transcription, DNA-templated; CC: nucleoplasm, DNA-directed RNA polymerase II, core complex; MF: transcription cofactor binding, DNA-dependent ATPase activity, DNA binding, chromatin binding, ATP binding, ATP-dependent helicase activity		0.669
USP46	64854	ubiquitin specific peptidase 46	BP: ubiquitin-dependent protein catabolic process, protein deubiquitination; MF: ubiquitin thiolesterase activity, protein binding, ubiquitin-specific protease activity		0.668
C10orf110	55853	chromosome 10 open reading frame 110			0.667
UNC45B	146862	unc-45 homolog B	BP: chaperone-mediated protein folding, cardiac muscle tissue development, cell differentiation, myofibril assembly; CC: cytosol, Z disc, A band; MF: Hsp90 protein binding		0.662
KPNA4	3840	karyopherin alpha 4 (importin alpha 3)	BP: protein import into nucleus, response to hydrogen peroxide, NLS-bearing protein import into nucleus, cytokine-mediated signaling pathway, protein transport; CC: nucleus, cytoplasm, extracellular vesicular exosome; MF: protein transporter activity, protein binding		0.655
SEMA4D	349236	Semaphorin-4D	BP: negative regulation of transcription from RNA polymerase II promoter, positive regulation of protein phosphorylation, negative regulation of cell adhesion, regulation of cell shape, negative regulation of alkaline phosphatase activity, positive regulation of phosphatidylinositol 3-kinase signaling, positive regulation of cell migration, positive regulation of GTPase activity, positive regulation of peptidyl-tyrosine phosphorylation, negative regulation of peptidyl-tyrosine phosphorylation; CC: extracellular space, plasma membrane; MF: receptor activity, transmembrane signaling receptor activity, receptor binding		0.654
GARNL3	84253	GTPase activating Rap/RanGAP domain-like 3	BP: positive regulation of GTPase activity, regulation of small GTPase mediated signal transduction; MF: GTPase activator activity		0.650
TJP1	7082	tight junction protein 1	BP: tight junction assembly, apoptotic process, cellular component disassembly involved in execution phase of apoptosis; CC: cytoplasm, plasma membrane, cell junction, tight junction, cytoplasmic vesicle, intercalated disc; MF: protein binding, protein domain specific binding	Adherens junction, Tight junction, Gap junction	0.645
LOC645550	645550				0.644
MACF1	23499	microtubule-actin crosslinking factor 1	BP: cell cycle arrest, metabolic process, Wnt signaling pathway, posttranslational protein targeting to membrane, establishment or maintenance of cell polarity, Golgi to plasma membrane protein transport; CC: cytoskeleton, Golgi apparatus; MF: actin binding, calcium ion binding, microtubule binding, ATPase activity, poly(A) RNA binding		0.642
ANO1	55107	anoctamin 1, calcium activated chloride channel	BP: regulation of membrane potential, cation transmembrane transport, chloride transmembrane transport; CC: external side of plasma membrane, integral component of membrane, extracellular exosome; MF: calcium activated cation channel activity, intracellular calcium activated chloride channel activity, protein homodimerization activity, protein heterodimerization activity		0.638
ZNF211	10520	zinc finger protein 211	BP: regulation of transcription, DNA-templated; CC: nucleus; MF: nucleic acid binding, metal ion binding, DNA binding		0.637
PAQR9	344838	progestin and adipoQ receptor family member IX	CC: integral component of membrane; MF: receptor activity		0.637
FYCO1	79443	FYVE and coiled-coil domain containing 1	BP: transport; CC: integral component of membrane		0.636
ARIH2	10425	ariadne RBR E3 ubiquitin protein ligase 2	BP: protein polyubiquitination, protein ubiquitination involved in ubiquitin-dependent protein catabolic process, developmental cell growth, protein K63-linked ubiquitination, protein K48-linked ubiquitination; CC: nucleus, cytoplasm; MF: ubiquitin-protein transferase activity, protein binding, zinc ion binding, nucleic acid binding, ligase activity		0.635
SLC30A1	7779	solute carrier family 30 (zinc transporter), member 1	BP: zinc II ion transport, cellular calcium ion homeostasis, negative regulation of calcium ion import, cellular zinc ion homeostasis, transmembrane transport, cadmium ion transmembrane transport; CC: cytoplasm, endoplasmic reticulum, Golgi apparatus, T-tubule, nuclear membrane, plasma membrane; MF: protein binding, calcium channel inhibitor activity, cation transmembrane transporter activity	Mineral absorption	0.635
SSH2	85464	slingshot homolog 2 protein phosphatase	BP: actin cytoskeleton organization, protein dephosphorylation, regulation of actin polymerization or depolymerization, regulation of axonogenesis; CC: cytoplasm, cytoskeleton; MF: DNA binding, protein tyrosine phosphatase activity, protein tyrosine/serine/threonine phosphatase activity, actin binding, phosphoprotein phosphatase activity	Regulation of actin cytoskeleton	0.628
HADHA	3030	hydroxyacyl-CoA dehydrogenase/3-ketoacyl-CoA thiolase/enoyl-CoA hydratase (trifunctional protein), alpha subunit	BP: fatty acid beta-oxidation, phospholipid metabolic process, cardiolipin acyl-chain remodeling, glycerophospholipid biosynthetic process, fatty acid metabolic process, oxidation-reduction process; CC: mitochondrion, mitochondrial inner membrane, mitochondrial fatty acid beta-oxidation multienzyme complex; MF: 3-hydroxyacyl-CoA dehydrogenase activity, acetyl-CoA C-acetyltransferase activity, enoyl-CoA hydratase activity, protein binding, long-chain-3-hydroxyacyl-CoA dehydrogenase activity, coenzyme binding, NAD binding	Fatty acid metabolism, Metabolic pathways, Fatty acid elongation, Fatty acid degradation, Biosynthesis of unsaturated fatty acids, Valine, leucine and isoleucine degradation, Lysine degradation, Tryptophan metabolism, beta-Alanine metabolism, Propanoate metabolism, Butanoate metabolism	0.624
CARKD	55739	carbohydrate kinase domain containing	BP: nicotinamide nucleotide metabolic process; CC: mitochondrion; MF: ATP binding, ATP-dependent NAD(P)H-hydrate dehydratase activity, lyase activity		0.623
DYRK2	8445	dual-specificity tyrosine-(Y)-phosphorylation regulated kinase 2	BP: intrinsic apoptotic signaling pathway in response to DNA damage by p53 class mediator, cellular response to DNA damage stimulus, protein phosphorylation, peptidyl-tyrosine phosphorylation, positive regulation of glycogen biosynthetic process, negative regulation of NFAT protein import into nucleus; CC: nucleus, cytoplasm, ubiquitin ligase complex, ribonucleoprotein complex; MF: magnesium ion binding, protein serine/threonine kinase activity, protein tyrosine kinase activity, ATP binding, ubiquitin binding		0.622
SPIRE1	56907	spire homolog 1	BP: Golgi vesicle transport, actin cytoskeleton organization, protein transport, actin nucleation; CC: Golgi apparatus, cytoplasmic vesicle membrane, cytoskeleton, Golgi apparatus, plasma membrane; MF: actin binding		0.621
FAM168B	130074	family with sequence similarity 168, member B	CC: extracellular vesicular exosome, plasma membrane, perinuclear region of cytoplasm, plasma membrane		0.611
PDE7B	27115	phosphodiesterase 7B	BP: cAMP-mediated signaling, signal transduction; CC: cytosol; MF: 3',5'-cyclic-AMP phosphodiesterase activity, metal ion binding, phosphoric diester hydrolase activity	Purine metabolism	0.610
CSGALNACT1	55790	chondroitin sulfate N-acetylgalactosaminyltransferase 1	BP: UDP-N-acetylgalactosamine metabolic process, extracellular matrix organization, UDP-glucuronate metabolic process, cartilage development; CC: Golgi cisterna membrane; MF: peptidoglycan glycosyltransferase activity, glucuronosyltransferase activity, glucuronylgalactosylproteoglycan 4-beta-N-acetylgalactosaminyltransferase activity	Glycosaminoglycan biosynthesis—chondroitin sulfate/dermatan sulfate, Metabolic pathways	0.609
MLLT10	8028	myeloid/lymphoid or mixed-lineage leukemia (trithorax homolog, Drosophila); translocated to, 10	BP: positive regulation of transcription from RNA polymerase II promoter, transcription, DNA-templated, canonical Wnt signaling pathway; CC: nucleus, cytoplasm; MF: zinc ion binding, DNA binding, sequence-specific DNA binding transcription factor activity		0.608
N4BP2L2	10443	NEDD4 binding protein 2-like 2	BP: negative regulation of transcription from RNA polymerase II promoter; CC: nucleus, transcriptional repressor complex, extracellular vesicular exosome, cytoplasm; MF: RNA polymerase II transcription corepressor activity, protein binding, enzyme binding		0.608
NARS	4677	asparaginyl-tRNA synthetase	BP:asparaginyl-tRNA aminoacylation, tRNA aminoacylation for protein translation, translation; CC: cytoplasm, mitochondrion, extracellular exosome; MF: nucleic acid binding, asparagine-tRNA ligase activity, aminoacyl-tRNA ligase activity, ATP binding	Aminoacyl-tRNA biosynthesis	0.606
LYSMD4	145748	LysM, putative peptidoglycan-binding, domain containing 4	CC: integral component of membrane, membrane		0.606
TXLNB	167838	taxilin beta	BP: positive regulation of neuron projection development; CC: cytoplasm; MF: syntaxin binding		0.604
TARBP1	6894	TAR (HIV-1) RNA binding protein 1	BP: regulation of transcription from RNA polymerase II promoter, RNA methylation, RNA processing; CC: nucleus; MF: RNA binding, RNA methyltransferase activity		0.601
FLJ23584	79640				0.596
SCAPER	49855	S-phase cyclin A-associated protein in the ER	CC: nucleus, cytoplasm, endoplasmic reticulum; MF: metal ion binding, nucleic acid binding		0.594
OBSL1	23363	obscurin-like 1	BP: microtubule cytoskeleton organization, Golgi organization, regulation of mitotic nuclear division, protein localization to Golgi apparatus, cardiac myofibril assembly; CC: Golgi apparatus, centrosome, cytoplasm, 3M complex; MF: cytoskeletal adaptor activity		0.585
SLC25A20	788	solute carrier family 25 (carnitine/acylcarnitine translocase), member 20	BP: cellular lipid metabolic process, transport, small molecule metabolic process; CC: mitochondrial inner membrane, mitochondrion		0.584
PPP3CB	5532	protein phosphatase 3, catalytic subunit, beta isozyme	CC: calcineurin complex, nucleus; MF: calcium channel inhibitor activity, calcium ion binding, calmodulin binding, enzyme binding, protein phosphatase 2B binding, phosphoprotein phosphatase activity, calmodulin-dependent protein phosphatase activity	MAPK signaling pathway, Calcium signaling pathway, cGMP-PKG signaling pathway, Apoptosis, Wnt signaling pathway, VEGF signaling pathway	0.581
ABCB4	5244	ATP-binding cassette, sub-family B (MDR/TAP), member 4	BP: transmembrane transport; CC: integral component of membrane, extracellular vesicular exosome, mitochondrion; MF: ATPase activity, coupled to transmembrane movement of substances, ATP binding, nucleotide binding, transporter activity	ABC transporters	0.580

BP: biological process; CC: cell component; MF: molecular function.

**Table 4:** Selected Signature Downregulated Gene Expressions in the Left Atria of Mitral Regurgitation vs. Normal Control

Symbol	Entrez ID	Gene Title	Gene Ontology	KEGG Pathway	Log_2_FC(MR/NC)
ITLN1	55600	intelectin 1 (galactofuranose binding)	BP: positive regulation of protein phosphorylation, positive regulation of glucose import; CC: anchored component of membrane, receptor complex, membrane raft, extracellular exosome; MF: carbohydrate binding		-5.119
NBPF20	400818	neuroblastoma breakpoint family, member 20, transcript variant 4	CC: cytoplasm		-2.848
TKT	7086	transketolase (Wernicke-Korsakoff syndrome)	BP: metabolic process; MF: catalytic activity	Carbon metabolism, Biosynthesis of amino acids, Metabolic pathways, Pentose phosphate pathway	-2.402
SFRP2	6423	secreted frizzled-related protein 2	BP: patterning of blood vessels, cardiac left ventricle morphogenesis, cell-cell signaling, response to nutrient, positive regulation of cell proliferation, negative regulation of gene expression, negative regulation of cardiac muscle cell apoptotic process, positive regulation of endopeptidase activity, negative regulation of Wnt signaling pathway, collagen fibril organization, positive regulation of cell growth, negative regulation of cell growth, negative regulation of cell migration, negative regulation of BMP signaling pathway, cellular response to extracellular stimulus, positive regulation of peptidyl-serine phosphorylation, positive regulation of cell adhesion mediated by integrin, positive regulation of catenin import into nucleus, non-canonical Wnt signaling pathway, positive regulation of apoptotic process, negative regulation of JUN kinase activity, negative regulation of transcription, DNA-templated, canonical Wnt signaling pathway, negative regulation of extrinsic apoptotic signaling pathway via death domain receptors, negative regulation of intrinsic apoptotic signaling pathway in response to DNA damage, negative regulation of planar cell polarity pathway involved in axis elongation; CC: extracellular space, extracellular matrix; MF: fibronectin binding, integrin binding, metalloenzyme activator activity, Wnt-protein binding, receptor agonist activity, endopeptidase activator activity	Wnt signaling pathway	-2.290
ARPC3	10094	actin related protein 2/3 complex, subunit 3	BP: movement of cell or subcellular component, Arp2/3 complex-mediated actin nucleation, Fc-gamma receptor signaling pathway involved in phagocytosis, ephrin receptor signaling pathway, actin filament organization; CC: cytosol, Arp2/3 protein complex, actin cytoskeleton, extracellular vesicular exosome, cell projection, lamellipodium; MF: structural constituent of cytoskeleton, protein binding, actin filament binding	Regulation of actin cytoskeleton, Fc gamma R-mediated phagocytosis	-2.124
SLCO2A1	6578	solute carrier organic anion transporter family, member 2A1	BP: lipid transport, prostaglandin transport, sodium-independent organic anion transport, transmembrane transport; CC: plasma membrane; MF: lipid transporter activity, prostaglandin transmembrane transporter activity		-2.115
LOC729841	729841				-1.872
NBPF10	440673	neuroblastoma breakpoint family, member 10, transcript variant 5	CC: cytoplasm; MF: poly(A) RNA binding		-1.783
RGS1	5996	regulator of G-protein signaling 1	BP: immune response, signal transduction, adenylate cyclase-inhibiting G-protein coupled receptor signaling pathway, termination of G-protein coupled receptor signaling pathway, positive regulation of GTPase activity; CC: cytoplasm, plasma membrane; MF: GTPase activator activity, calmodulin binding		-1.764
ADH1B	125	alcohol dehydrogenase IB (class I), beta polypeptide	BP: oxidation-reduction process; MF: zinc ion binding, oxidoreductase activity	Metabolic pathways, Glycolysis/Gluconeogenesis, Fatty acid degradation, Tyrosine metabolism	-1.727
APOE	348	apolipoprotein E	BP: cholesterol biosynthetic process, cholesterol catabolic process, triglyceride catabolic process, cholesterol transport, lipoprotein catabolic process, neuron projection regeneration; CC: extracellular region; MF: cholesterol binding, cholesterol transporter activity		-1.716
RPS3	6188	ribosomal protein S3	BP: DNA catabolic process, endonucleolytic, cytoplasmic translation, DNA repair; CC: ribosome, nucleus, cytosolic small ribosomal subunit; MF: structural constituent of ribosome, RNA binding, damaged DNA binding, oxidized purine nucleobase lesion DNA N-glycosylase activity, protein kinase A binding	Ribosome	-1.604
LOC388654	388654				-1.477
LOC651149	651149				-1.434
LOC390354	390354				-1.422
LOC729926	729926				-1.399
C8orf4	56892	chromosome 8 open reading frame 4	BP: apoptotic process		-1.397
RARRES2	5919	retinoic acid receptor responder (tazarotene induced) 2	BP: inflammatory response, regulation of lipid catabolic process; MF: receptor binding		-1.310
TMBIM1	64114	transmembrane BAX inhibitor motif containing 1	BP: negative regulation of extrinsic apoptotic signaling pathway via death domain receptors, negative regulation of Fas signaling pathway, negative regulation of establishment of protein localization to plasma membrane; CC: integral component of membrane, Golgi apparatus, lysosomal membrane, extracellular vesicular exosome; MF: death receptor binding		-1.295
NBPF8	728841	neuroblastoma breakpoint family, member 8	CC: cytoplasm		-1.281
PPP3R1	5534	protein phosphatase 3 (formerly 2B), regulatory subunit B, alpha isoform, Calcineurin subunit B type 1	BP: stimulatory C-type lectin receptor signaling pathway, apoptotic process, dephosphorylation, calcineurin-NFAT signaling cascade, Fc-epsilon receptor signaling pathway, innate immune response, positive regulation of transcription from RNA polymerase II promoter, positive regulation of NFAT protein import into nucleus, intrinsic apoptotic signaling pathway, positive regulation of protein insertion into mitochondrial membrane involved in apoptotic signaling pathway; CC: sarcolemma, nucleoplasm, cytosol, calcineurin complex; MF: calcium ion binding, calcium-dependent protein serine/threonine phosphatase activity, calcium ion binding, protein binding, calmodulin binding, protein domain specific binding	MAPK signaling pathway, Calcium signaling pathway, cGMP-PKG signaling pathway, Apoptosis, Wnt signaling pathway, VEGF signaling pathway, Glucagon signaling pathway	-1.267
DDOST	1650	dolichyl-diphosphooligosaccharide-protein glycosyltransferase	BP: protein N-linked glycosylation via asparagine, cellular protein metabolic process, gene expression, post-translational protein modification, protein glycosylation, translation; CC: endoplasmic reticulum membrane, intracellular membrane-bounded organelle; MF: dolichyl-diphosphooligosaccharide-protein glycotransferase activity, oligosaccharyl transferase activity, protein binding	Metabolic pathways, N-Glycan biosynthesis, Protein processing in endoplasmic reticulum	-1.264
ZFYVE21	79038	zinc finger, FYVE domain containing 21	CC: endosome, focal adhesion, cytoplasmic membrane-bounded vesicle; MF: protein binding, metal ion binding		-1.255
PDIA3P	171423	protein disulfide isomerase family A, member 3 pseudogene			-1.245
LOC441131	441131				-1.237
LOC654194	654194				-1.234
SLC39A8	64116	solute carrier family 39 (zinc transporter), member 8	BP: transmembrane transport, zinc ion transport; CC: integral component of membrane; MF: metal ion transmembrane transporter activity		-1.230
TAGLN	6876	transgelin	BP: muscle organ development, epithelial cell differentiation; CC: cytoplasm; MF: actin filament binding, protein binding		-1.223
FKBP5	2289	FK506 binding protein 5	BP: protein peptidyl-prolyl isomerization, protein folding, chaperone-mediated protein folding; CC: nucleoplasm, cytoplasm, endoplasmic reticulum membrane, extracellular exosome; MF: peptidyl-prolyl cis-trans isomerase activity, protein binding, FK506 binding, heat shock protein binding	Estrogen signaling pathway	-1.213
LOC648024	648024				-1.193
LOC387867	387867				-1.182
LOC100131905	100131905				-1.139
PHGDH	26227	phosphoglycerate dehydrogenase	BP: cell cycle process, cellular amino acid biosynthetic process, cellular nitrogen compound metabolic process, glutamine metabolic process, regulation of gene expression, small molecule metabolic process, taurine metabolic process, threonine metabolic process, L-serine biosynthetic process; CC: cytosol; MF: electron carrier activity, NAD binding, phosphoglycerate dehydrogenase activity	Biosynthesis of amino acids, Carbon metabolism, Glycine, serine and threonine metabolism, Metabolic pathways	-1.132
LOC728139	728139				-1.126
LOC728698	728698				-1.107
LOC284821	284821				-1.106
EPHX1	2052	epoxide hydrolase 1, microsomal	BP: cellular aromatic compound metabolic process; CC: endoplasmic reticulum membrane; MF: cis-stilbene-oxide hydrolase activity, epoxide hydrolase activity		-1.081
PICALM	8301	phosphatidylinositol binding clathrin assembly protein	BP: clathrin coat assembly; CC: clathrin coat, intracellular membrane-bounded organelle; MF: 1-phosphatidylinositol binding, clathrin binding, phospholipid binding		-1.081
CFD	1675	complement factor D	BP: proteolysis, complement activation, blood coagulation, platelet degranulation, platelet activation; CC: extracellular region, platelet alpha granule lumen extracellular exosome; MF: serine-type endopeptidase activity	Complement and coagulation cascades	-1.072
GAS1	2619	growth arrest-specific 1	BP: regulation of smoothened signaling pathway, negative regulation of protein processing, cellular response to vascular endothelial growth factor stimulus, regulation of apoptotic process, cell fate commitment, negative regulation of mitotic cell cycle, developmental growth, regulation of ER to Golgi vesicle-mediated transport, positive regulation of mesenchymal cell proliferation, cell cycle arrest, negative regulation of cell growth, positive regulation of epithelial cell proliferation, negative regulation of epithelial cell proliferation, negative regulation of extrinsic apoptotic signaling pathway in absence of ligand; CC: plasma membrane, integral component of membrane; MF: protein binding	Hedgehog signaling pathway	-1.067
FKBP2	2286	FK506 binding protein 2	BP: protein folding, peptidyl-proline modification;CC: endoplasmic reticulum, membrane; MF: peptidyl-prolyl cis-trans isomerase activity, FK506 binding, protein binding		-1.058
RPL21	6144	ribosomal protein L21	BP: translation;CC: nucleolus, cytoplasm, ribosome; MF: structural constituent of ribosome, poly(A) RNA binding	Ribosome	-1.044
RNASE4	6038	ribonuclease, RNase A family, 4	BP: mRNA cleavage, RNA phosphodiester bond hydrolysis, endonucleolytic; CC: extracellular region, extracellular exosome; MF: nucleic acid binding, ribonuclease A activity		-1.039
OAZ2	4947	ornithine decarboxylase antizyme 2	BP: cellular nitrogen compound metabolic process, negative regulation of catalytic activity, polyamine metabolic process, regulation of cellular amino acid metabolic process, small molecule metabolic process; CC: cytosol, nucleus; MF: ornithine decarboxylase inhibitor activity		-1.025
MAGOH	4116	mago-nashi homolog, proliferation-associated	BP: nuclear-transcribed mRNA catabolic process, nonsense-mediated decay, regulation of alternative mRNA splicing, via spliceosome, mRNA splicing, via spliceosome, transcription from RNA polymerase II promoter, termination of RNA polymerase II transcription, mRNA export from nucleus, regulation of translation, RNA splicing, gene expression, mRNA 3'-end processing; CC: nucleus, nucleoplasm, cytosol, nuclear speck, exon-exon junction complex, catalytic step 2 spliceosome; MF: protein binding, poly(A) RNA binding	RNA transport, mRNA surveillance pathway, Spliceosome	-1.019
LOC648249	648249				-1.004
PTPRF	5792	protein tyrosine phosphatase, receptor type, F	BP: peptidyl-tyrosine dephosphorylation, cell adhesion, transmembrane receptor protein tyrosine phosphatase signaling pathway, cell migration, negative regulation of receptor binding; CC: integral component of plasma membrane, extracellular exosome; MF: protein tyrosine phosphatase activity, transmembrane receptor protein tyrosine phosphatase activity, protein complex binding	Cell adhesion molecules, Adherens junction, Insulin signaling pathway	-0.997
LOC441775	441775				-0.991
LOC440055	440055				-0.990
LOC100129553	100129553				-0.978
RAB32	10981	RAB32, member RAS oncogene family	BP: intracellular protein transport, metabolic process, Rab protein signal transduction, endosome to melanosome transport, phagosome maturation; CC: mitochondrion, early endosome, trans-Golgi network, membrane, phagocytic vesicle membrane, melanosome; MF: GTPase activity, protein binding, GTP binding, GTP-dependent protein binding, AP-1 adaptor complex binding, AP-3 adaptor complex binding, BLOC-2 complex binding		-0.965
CHST7	56548	carbohydrate (N-acetylglucosamine 6-O) sulfotransferase 7	BP: carbohydrate metabolic process, polysaccharide metabolic process, N-acetylglucosamine metabolic process, sulfur compound metabolic process, glycosaminoglycan metabolic process, chondroitin sulfate metabolic process; CC: Golgi membrane, integral component of membrane; MF: N-acetylglucosamine 6-O-sulfotransferase activity, chondroitin 6-sulfotransferase activity	Glycosaminoglycan biosynthesis—chondroitin sulfate / dermatan sulfate	-0.961
DHCR24	1718	24-dehydrocholesterol reductase	BP: cholesterol biosynthetic process, apoptotic process, negative regulation of apoptotic process, negative regulation of cysteine-type endopeptidase activity involved in apoptotic process, response to oxidative stress, oxidation-reduction process, cell cycle arrest, Ras protein signal transduction, protein localization, negative regulation of cell proliferation, plasminogen activation, amyloid precursor protein catabolic process; CC: Golgi membrane, nucleus, cytoplasm, endoplasmic reticulum, cytoskeleton; MF: delta24(24–1) sterol reductase activity, UDP-N-acetylmuramate dehydrogenase activity, oxidoreductase activity, acting on the CH-CH group of donors, NAD or NADP as acceptor, enzyme binding, peptide antigen binding, flavin adenine dinucleotide binding	Steroid biosynthesis, Metabolic pathways	-0.952
GCHFR	2644	GTP cyclohydrolase I feedback regulator	BP: negative regulation of biosynthetic process, negative regulation of GTP cyclohydrolase I activity; CC: cytoplasm, nucleus, protein complex; MF: amino acid binding, enzyme inhibitor activity, GTP cyclohydrolase binding, GTP-dependent protein binding, hydrolase activity		-0.939
LOC643319	643319				-0.939
DCN	1634	decorin	MF: collagen binding	TGF-beta signaling pathway	-0.936
LOC653079	653079				-0.927
DNCL1	8655	dynein, cytoplasmic, light polypeptide 1	BP: transcription, DNA-templated, regulation of transcription, DNA-templated, transport, microtubule-based process, regulation of catalytic activity, G2/M transition of mitotic cell cycle, mitotic cell cycle, apoptotic process, organelle organization, actin cytoskeleton organization, negative regulation of phosphorylation, positive regulation of protein insertion into mitochondrial membrane involved in apoptotic signaling pathway, intrinsic apoptotic signaling pathway; CC: mitochondrion, microtubule, nucleus, cytoplasm, cytoplasmic dynein complex, extracellular exosome, mitotic spindle, COP9 signalosome; MF: motor activity, enzyme binding, nitric-oxide synthase regulator activity, protein homodimerization activity, protein binding		-0.922
SEPP1	6414	selenoprotein P, plasma, 1	BP: selenium compound metabolic process, growth, response to oxidative stress; CC: extracellular space, extracellular exosome; MF: selenium binding		-0.917
MAFB	9935	v-maf musculoaponeurotic fibrosarcoma oncogene homolog B	BP: positive regulation of transcription from RNA polymerase II promoter, transcription, DNA-templated; CC: nucleus, transcription factor complex; MF: sequence-specific DNA binding transcription factor activity, sequence-specific DNA binding, transcription factor binding, DNA binding		-0.910
LOC441506	441506				-0.905
NME1-NME2	654364	NME1-NME2 readthrough	BP: nucleoside diphosphate phosphorylation, GTP biosynthetic process, negative regulation of gene expression, regulation of apoptotic process; CC: centrosome, cytosol, mitochondrion; MF: RNA polymerase II regulatory region sequence-specific DNA binding, single-stranded DNA binding, nucleoside diphosphate kinase activity, ATP binding, protein kinase binding	Metabolic pathways, Purine metabolism, Pyrimidine metabolism	-0.905
CXCR7	57007	chemokine (C-X-C motif) receptor 7	BP: angiogenesis, vasculogenesis, cell adhesion, G-protein coupled receptor signaling pathway, receptor internalization, chemokine-mediated signaling pathway, positive regulation of ERK1 and ERK2 cascade, negative regulation of intrinsic apoptotic signaling pathway in response to DNA damage, chemotaxis; CC: endosome, plasma membrane, perinuclear region of cytoplasm; MF: scavenger receptor activity, coreceptor activity, C-X-C chemokine receptor activity, signal transducer activity		-0.903
LOC653881	653881				-0.887
SIVA1	10572	SIVA1, apoptosis-inducing factor	BP: negative regulation of NF-kappaB transcription factor activity, extrinsic apoptotic signaling pathway, intrinsic apoptotic signaling pathway, positive regulation of mitochondrial outer membrane permeabilization involved in apoptotic signaling pathway; CC: cytoplasm, nucleoplasm, mitochondrion; MF: CD27 receptor binding, zinc ion binding, tumor necrosis factor receptor binding		-0.863
LOC286444	286444				-0.860
LOC100128892	100128892				-0.859
LOC728658	728658				-0.859
LOC401537	401537				-0.837
C2orf40	84417	chromosome 2 open reading frame 40	BP: negative regulation of cyclin-dependent protein serine/threonine kinase by cyclin degradation, G1 to G0 transition, cellular senescence; CC: extracellular space, transport vesicle		-0.820
LOC649049	649049				-0.812
C13orf15	28984	chromosome 13 open reading frame 15	BP: negative regulation of exit from mitosis, negative regulation of endothelial cell proliferation, positive regulation of extracellular matrix constituent secretion, complement activation, positive regulation of epithelial to mesenchymal transition, negative regulation of angiogenesis, positive regulation of cyclin-dependent protein serine/threonine kinase activity involved in G1/S transition of mitotic cell cycle, positive regulation of collagen biosynthetic process, positive regulation of mitotic nuclear division, positive regulation of transcription from RNA polymerase II promoter, negative regulation of cytokine secretion, positive regulation of cytokine secretion, positive regulation of sequence-specific DNA binding transcription factor activity, positive regulation of stress fiber assembly, positive regulation of cell cycle arrest, mitotic cell cycle arrest, negative regulation of mitotic cell cycle phase transition; CC: nucleus, cytoplasm, centrosome; MF: protein kinase activator activity, R-SMAD binding		-0.809
IRF2BP2	359948	interferon regulatory factor 2 binding protein 2	BP: transcription, DNA-templated, regulation of transcription, DNA-templated; CC: nucleus, cytoplasm; MF: metal ion binding		-0.805
LOC148430	148430				-0.803
LOC641814	641814				-0.779
RPS5	6193	ribosomal protein S5	BP: translation, nuclear-transcribed mRNA catabolic process, nonsense-mediated decay, translational initiation, translational elongation, translational termination, SRP-dependent cotranslational protein targeting to membrane, gene expression, cellular protein metabolic process; CC: small ribosomal subunit, cytosol, focal adhesion, ribonucleoprotein complex, extracellular exosome; MF: RNA binding, structural constituent of ribosome, poly(A) RNA binding		-0.772
PGRMC1	10857	progesterone receptor membrane component 1	CC: endoplasmic reticulum membrane, integral component of membrane, nucleolus, extracellular exosome; MF: steroid binding, protein binding, heme binding		-0.771
ANXA2	302	annexin A2	BP: negative regulation of catalytic activity; MF: phospholipase inhibitor activity, calcium ion binding, calcium-dependent phospholipid binding, cytoskeletal protein binding		-0.768
TNFRSF21	27242	tumor necrosis factor receptor superfamily, member 21	BP: adaptive immune response, apoptotic process, cellular, cellular response to tumor necrosis factor, humoral immune response, myelination, T cell receptor signaling pathway, negative regulation of interleukin-10 secretion, negative regulation of interleukin-13 secretion, negative regulation of interleukin-5 secretion; CC: integral component of plasma membrane, plasma membrane; MF: protein binding	Cytokine-cytokine receptor interaction	-0.767
PALM	5064	paralemmin	BP: regulation of cell shape, movement of cell or subcellular component, negative regulation of adenylate cyclase activity, positive regulation of filopodium assembly, cytoskeleton organization, negative regulation of dopamine receptor signaling pathway, protein targeting to plasma membrane; CC: membrane, nucleus, nucleoplasm, plasma membrane, cytoplasmic membrane-bounded vesicle; MF: protein binding, D3 dopamine receptor binding		-0.767
SLC25A6	293	solute carrier family 25 (mitochondrial carrier; adenine nucleotide translocator), member 6	BP: apoptotic process, transmembrane transport, protein targeting to mitochondrion, ADP transport, ATP transport, cellular protein metabolic process; CC: nucleus, mitochondrial inner membrane, integral component of membrane; MF: transporter activity, ATP:ADP antiporter activity, protein binding	Calcium signaling pathway, cGMP-PKG signaling pathway	-0.764
LOC652624	652624				-0.760
CKLF	51192	chemokine-like factor	BP: cell proliferation, neutrophil chemotaxis, secretion by cell, macrophage chemotaxis, lymphocyte chemotaxis; CC: integral component of membrane, extracellular space; MF: chemokine activity		-0.756
LOC642741	642741				-0.755
LOC730187	730187				-0.746
SLC9A3R1	9368	solute carrier family 9, subfamily A (NHE3, cation proton antiporter 3), member 3 regulator 1	BP: adenylate cyclase-activating dopamine receptor signaling pathway, negative regulation of cell proliferation, negative regulation of platelet-derived growth factor receptor signaling pathway, negative regulation of phosphatidylinositol 3-kinase signaling, actin cytoskeleton organization, negative regulation of cell migration, negative regulation of sodium:proton antiporter activity, negative regulation of protein kinase B signaling, glutathione transport, cellular protein localization, phospholipase C-activating dopamine receptor signaling pathway, negative regulation of ERK1 and ERK2 cascade, positive regulation of intrinsic apoptotic signaling pathway, negative regulation of phosphatidylinositol 3-kinase signaling; CC: cytoplasm, intracellular membrane-bounded organelle, centrosome; MF: beta-catenin binding, chloride channel regulator activity, phosphatase binding, PDZ domain binding, beta-2 adrenergic receptor binding, dopamine receptor binding, growth factor receptor binding		-0.745
CES1	1066	carboxylesterase 1 (monocyte/macrophage serine esterase 1), transcript variant 5	BP: metabolic process, epithelial cell differentiation; CC: endoplasmic reticulum lumen; MF: methylumbelliferyl-acetate deacetylase activity, carboxylic ester hydrolase activity	Metabolic pathways	-0.745
LOC646785	646785				-0.720
TSPAN4	7106	tetraspanin 4	BP: protein complex assembly; CC: plasma membrane, focal adhesion; MF: antigen binding, integrin binding		-0.718
SSR2	6746	signal sequence receptor, beta (translocon-associated protein beta)	BP: chromatin remodeling, regulation of transcription from RNA polymerase II promoter, translation, SRP-dependent cotranslational protein targeting to membrane, gene expression, cellular protein metabolic process; CC: endoplasmic reticulum, integral component of membrane, phagocytic vesicle, SWI/SNF complex, RSC complex, cytoplasm; MF: DNA binding, zinc ion binding	Protein processing in endoplasmic reticulum	-0.715
RPL12	6136	ribosomal protein L12	BP: nuclear-transcribed mRNA catabolic process, nonsense-mediated decay, translation, translational initiation, translational elongation, translational termination, SRP-dependent cotranslational protein targeting to membrane, gene expression, cellular protein metabolic process; CC: cytosol, focal adhesion, cytosolic large ribosomal subunit, extracellular exosome; MF: structural constituent of ribosome, protein binding, poly(A) RNA binding	Ribosome	-0.709
MS4A7	58475	membrane-spanning 4-domains, subfamily A, member 7	CC: integral component of membrane		-0.709
TSPO	706	translocator protein	BP: positive regulation of apoptotic process, positive regulation of necrotic cell death, negative regulation of nitric oxide biosynthetic process, positive regulation of reactive oxygen species metabolic process, regulation of oxidative phosphorylation, positive regulation of mitochondrial depolarization, negative regulation of tumor necrosis factor production, steroid biosynthetic process, chloride transport, positive regulation of calcium ion transport; CC: mitochondrial envelope, mitochondrial outer membrane, integral component of membrane	Neuroactive ligand-receptor interaction	-0.708
LOC731096	731096				-0.705
LOC387930	387930				-0.704
PPDPF	79144	pancreatic progenitor cell differentiation and proliferation factor	BP: multicellular organismal development, cell differentiation		-0.700
LOC441013	441013				-0.699
LOC647436	647436				-0.699
ATF5	22809	activating transcription factor 5	BP: regulation of transcription from RNA polymerase II promoter, transcription from RNA polymerase II promoter, negative regulation of nucleic acid-templated transcription, negative regulation of apoptotic process; CC: nucleoplasm, cytoplasm, transcription factor complex; MF: sequence-specific DNA binding transcription factor activity, RNA polymerase II transcription regulatory region sequence-specific DNA binding transcription factor activity involved in positive regulation of transcription, transcription corepressor activity, protein binding, heat shock protein binding		-0.694
APBB1IP	54518	amyloid beta (A4) precursor protein-binding, family B, member 1 interacting protein	BP: signal transduction, positive regulation of cell adhesion; CC: cytoplasm, cytoskeleton, plasma membrane, cell junction	Rap1 signaling pathway, Platelet activation	-0.691
LMCD1	29995	LIM and cysteine-rich domains 1	BP: positive regulation of calcineurin-NFAT signaling cascade, negative regulation of nucleic acid-templated transcription, regulation of cardiac muscle hypertrophy, activation of mitophagy in response to mitochondrial depolarization, negative regulation of transcription from RNA polymerase II promoter, transcription, DNA-templated; CC: nucleus, extracellular space, extracellular matrix; MF: transcription corepressor activity, zinc ion binding		-0.683
LOC648294	648294				-0.680
AVPI1	60370	arginine vasopressin-induced 1	BP: activation of MAPK activity, cell cycle; MF: protein binding		-0.669
LOC729679	729679				-0.665
LOC645387	645387				-0.665
ENSA	2029	endosulfine alpha	BP: G2/M transition of mitotic cell cycle, mitotic cell cycle, transport, mitotic nuclear division, regulation of protein phosphatase type 2A activity, negative regulation of catalytic activity, cell division; CC: nucleoplasm, cytoplasm; MF: receptor binding, ion channel inhibitor activity, protein phosphatase type 2A regulator activity, phosphatase inhibitor activity, potassium channel inhibitor activity, protein phosphatase 2A binding		-0.662
LOC728128	728128				-0.652
BTG3	10950	BTG family, member 3	BP: negative regulation of cell proliferation, negative regulation of mitotic cell cycle; CC: cytoplasm; MF: protein binding	RNA degradation	-0.650
UNG	7374	uracil-DNA glycosylase	BP: negative regulation of apoptotic process, DNA repair, base-excision repair, depyrimidination; CC: mitochondrion, nucleus, nucleoplasm; MF: uracil DNA N-glycosylase activity, protein binding		-0.647
CNN2	1265	calponin 2	BP: actomyosin structure organization, positive regulation of gene expression, negative regulation of cell migration, regulation of cell proliferation, cytoskeleton organization, cellular response to mechanical stimulus, regulation of actin filament-based process; CC: cytoskeleton, stress fiber, cell-cell junction, focal adhesion, extracellular exosome; MF: actin binding, calmodulin binding		-0.641
GJC2	57165	gap junction protein, gamma 2	BP: cell death, cell-cell signaling, response to toxic substance, transmembrane transport; CC: connexon complex, integral component of membrane; MF: gap junction channel activity		-0.639
PGLS	25796	6-phosphogluconolactonase	BP: carbohydrate metabolic process, pentose-phosphate shunt; CC: cytosol, extracellular vesicular exosome; MF: 6-phosphogluconolactonase activity, monosaccharide binding	Carbon metabolism, Pentose phosphate pathway, Metabolic pathways	-0.639
AGTR1	185	angiotensin II receptor, type 1	BP: angiotensin-activated signaling pathway, Rho protein signal transduction, positive regulation of cholesterol esterification, regulation of vasoconstriction, calcium-mediated signaling, positive regulation of cellular protein metabolic process, positive regulation of phospholipase A2 activity, positive regulation of cytosolic calcium ion concentration involved in phospholipase C-activating G-protein coupled signaling pathway, phospholipase C-activating angiotensin-activated signaling pathway, regulation of cell growth; CC: plasma membrane, integral component of membrane; MF: angiotensin type II receptor activity, angiotensin type I receptor activity, bradykinin receptor binding	Vascular smooth muscle contraction, Renin-angiotensin system, Calcium signaling pathway, cGMP-PKG signaling pathway, Neuroactive ligand-receptor interaction, Adrenergic signaling in cardiomyocytes	-0.636
LOC729798	729798				-0.635
CAMK1	8536	calcium/calmodulin-dependent protein kinase I	BP: cell cycle, positive regulation of muscle cell differentiation, positive regulation of protein export from nucleus, protein phosphorylation, regulation of protein binding, regulation of protein localization, signal transduction, nucleocytoplasmic transport; CC: cytoplasm, nucleus; MF: ATP binding, protein serine/threonine kinase activity, calmodulin binding, calmodulin-dependent protein kinase activity, protein binding		-0.627
APRT	353	adenine phosphoribosyltransferase	BP: purine-containing compound salvage, purine ribonucleoside salvage, nucleoside metabolic process; CC: cytoplasm; MF: adenine phosphoribosyltransferase activity, hypoxanthine phosphoribosyltransferase activity, AMP binding, transferase activity, transferring glycosyl groups	Purine metabolism, Metabolic pathways	-0.625
SFRS7	6432	splicing factor, arginine/serine-rich 7	BP: mRNA processing, mRNA export from nucleus, RNA splicing, mRNA splicing, via spliceosome, transcription from RNA polymerase II promoter, termination of RNA polymerase II transcription, negative regulation of mRNA splicing, via spliceosome, gene expression, mRNA 3'-end processing; CC: nucleoplasm, cytoplasm, extracellular exosome; MF: nucleic acid binding, zinc ion binding, nucleotide binding, poly(A) RNA binding, protein binding		-0.624
LOC653232	653232				-0.618
TUBB	203068	tubulin, beta	BP: metabolic process, spindle assembly, protein polymerization; CC: nuclear envelope lumen, cytoplasmic ribonucleoprotein granule, extracellular exosome; MF: GTP binding, structural constituent of cytoskeleton, GTPase activity, ubiquitin protein ligase binding	Phagosome, Gap junction	-0.617
ERGIC3	51614	ERGIC and golgi 3	BP: vesicle-mediated transport; CC: endoplasmic reticulum membrane, Golgi apparatus, endoplasmic reticulum-Golgi intermediate compartment membrane		-0.617
LOC644315	644315				-0.608
RPL26	6154	ribosomal protein L26	BP: translation, nuclear-transcribed mRNA catabolic process, nonsense-mediated decay, rRNA processing, translational initiation, translational elongation, translational termination, SRP-dependent cotranslational protein targeting to membrane, gene expression, cellular protein metabolic process; CC: large ribosomal subunit, cytosol, extracellular exosome; MF: structural constituent of ribosome, RNA binding, poly(A) RNA binding	Ribosome	-0.604
DHRS3	9249	dehydrogenase/reductase (SDR family) member 3	BP: oxidation-reduction process, cardiac septum morphogenesis, retinoid metabolic process; CC: endoplasmic reticulum membrane; MF: oxidoreductase activity	Metabolic pathways	-0.603
SEC11C	90701	SEC11 homolog C	BP: signal peptide processing, proteolysis, translation, SRP-dependent cotranslational protein targeting to membrane, gene expression, cellular protein metabolic process; CC: integral component of membrane, endoplasmic reticulum membrane; MF: serine-type peptidase activity, hydrolase activity	Protein export	-0.600
LOC643358	643358				-0.598
TIGA1	114915	TIGA1			-0.598
LOC285900	285900				-0.596
EIF4A1	1973	eukaryotic translation initiation factor 4A, isoform 1	BP: metabolic process; CC: extracellular vesicular exosome, nucleus, cytoplasm; MF: nucleic acid binding, helicase activity, ATP-dependent helicase activity, ATP binding, double-stranded RNA binding, translation initiation factor activity, poly(A) RNA binding	RNA transport	-0.591
LOC388720	388720				-0.587
ADH1C	126	alcohol dehydrogenase 1C (class I), gamma polypeptide	BP: oxidation-reduction process, small molecule metabolic process; CC: cytosol; MF: alcohol dehydrogenase (NAD) activity, oxidoreductase activity, zinc ion binding	Metabolic pathways, Glycolysis / Gluconeogenesis, Fatty acid degradation, Tyrosine metabolism, Drug metabolism, Retinol metabolism	-0.587
SEC61B	10952	Sec61 beta subunit	BP: intracellular protein transport, protein import into nucleus, translocation, antigen processing and presentation of peptide antigen via MHC class I, translation, SRP-dependent cotranslational protein targeting to membrane, gene expression, ER-associated ubiquitin-dependent protein catabolic process, endoplasmic reticulum unfolded protein response, retrograde protein transport, ER to cytosol, IRE1-mediated unfolded protein response, cellular protein metabolic process; CC: Sec61 translocon complex, endoplasmic reticulum, cytosol, endoplasmic reticulum Sec complex; MF: protein binding, poly(A) RNA binding, epidermal growth factor binding	Protein processing in endoplasmic reticulum, Protein export, Phagosome	-0.583

BP: biological process; CC: cell component; MF: molecular function.

**Fig 2:**
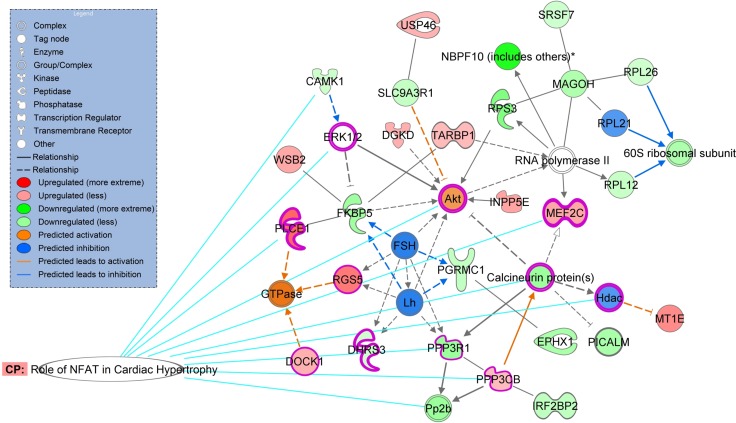
The network with highest score (*P*-score = 50, i.e. *P*-value < 10^−50^) was derived from 244 differentially expressed genes using Ingenuity Pathway Analysis Global Molecular Network algorithm. The edge in this network represents a relationship between two genes based on Ingenuity Pathways Knowledge Base. The genes with violet border color represent its functions related to cardiovascular system development, such as MEF2C, PLCE1, PPP3CB, and PPP3R1.

To elucidate the molecular mechanisms of MR on left atrial gene expression, we used Ingenuity Pathway Analysis to search for enrichment in predicted function. A network with highest score (*P*-score = 50, i.e. *P* value < 10^−50^) was generated from 244 differentially expressed genes using Ingenuity Pathway Analysis Global Molecular Network algorithm as depicted in Fig 2, and 26 focused genes were identified to be involved in the network, including PLCE1, PPP3R1, PPP3CB, MEF2C and etc. Top involved canonical pathways in this network included role of nuclear factor of activated T cells (NFAT) in cardiac hypertrophy, cardiac hypertrophy signaling, and calcium signaling (Table 5). Top diseases and functions in this network included cardiovascular system development and function, organ morphology, and organismal development (Table 5). These results demonstrated that the network was significantly associated with cardiac related pathways and functions, such as role of NFAT in cardiac hypertrophy and cardiovascular system development and function.

**Table 5:** Top Involved Canonical Pathways and Top Diseases and Functions in the Network Derived from 244 Differentially Expressed Genes between Mitral Regurgitation Patients and Normal Subjects Using Ingenuity Pathway Analysis Global Molecular Network Algorithm

Top Canonical Pathways and Diseases and Functions [Genes]	*P* Value
Role of NFAT in cardiac hypertrophy [PPP3R1, PPP3CB, Calcineurin protein(s), PLCE1, Hdac, ERK1/2, Akt, Pp2b, CAMK1, MEF2C]	4.03E-06
Cardiac hypertrophy signaling [PPP3R1, PPP3CB, Calcineurin protein(s), PLCE1, ERK1/2, Akt, Pp2b, MEF2C]	2.33E-04
Calcium signaling [PPP3R1, PPP3CB, Calcineurin protein(s), Hdac, ERK1/2, Pp2b, CAMK1, MEF2C]	8.64E-05
Cardiovascular system development and function [PPP3R1, PPP3CB, Calcineurin protein(s), PLCE1, Hdac, DOCK1, DHRS3, PICALM, ERK1/2, Akt, RGS5, MEF2C]	3.1E-6–8.08E-2
Organ morphology [PPP3R1, PPP3CB, Calcineurin protein(s), PLCE1, Hdac, DOCK1, DHRS3, PICALM, ERK1/2, Akt, INPP5E, MEF2C]	3.1E-6–4.67E-2
Organismal development [PPP3R1, PPP3CB, Calcineurin protein(s), PLCE1, Hdac, DOCK1, DHRS3, PICALM, INPP5E, SLC9A3R1, MEF2C]	3.1E-6–9.22E-2

NFAT = nuclear factor of activated T cells.

**Fig 3:**
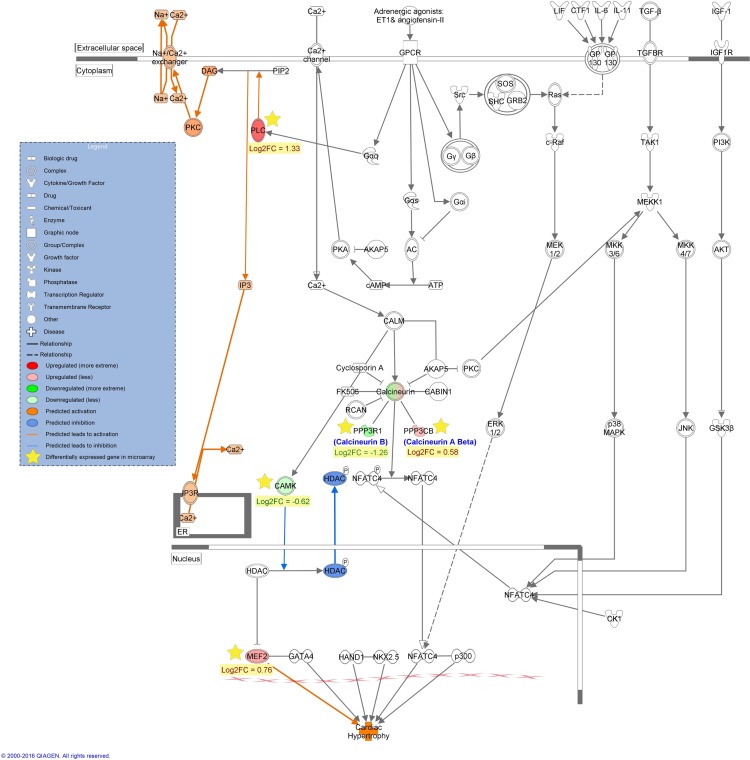
The “Role of NFAT in cardiac hypertrophy” pathway.

Furthermore, we applied the activation z-score analysis method, which was proposed by Andreas Krämer et al [5] in 2014, to measure activation states (increased or decreased) of the pathways affected by differentially expressed genes. We take a statistical approach by defining a quantity (z‐score) that determines whether a biological function has significantly more “increased” predictions than “decreased” predictions (z>0) or vice versa (z<0). In practice, z‐scores greater than 2 or smaller than ‐2 can be considered significant. Only “Role of NFAT in cardiac hypertrophy” pathway (Fig 3) had a z-score of 1.34 and *P* < 0.02. Thus, according to log2 fold-change values and the predictive activities of the differentially expressed genes significantly involved in NFAT pathway (Table 6), we derived an activation z-score equal to 1.34 which suggests that these differentially expressed genes moderately activate the Role of NFAT in cardiac hypertrophy pathway (Fig 3). The detailed information of differentially expressed genes involved in NFAT pathway was shown in Table 6, implicating that the expression patterns of PPP3CB (Calcineurin A beta), PPP3R1 (Calcineurin B), PLCE1, MEF2C, and CAMK1 played a role in the activation of hypertrophy of atrial myocytes in MR patients compared to normal subjects. The results of the real-time quantitative RT-PCR of these 5 genes were consistent with the RNA microarray data (Table 7).

**Table 6:** Log2 Fold Change Values and Predictive Activity of the Differentially Expressed Genes Significantly Involved in Role of NFAT in Cardiac Hypertrophy Pathway

Symbol	Entrez Gene Name	Log2FC value	Predictive Activity to Pathway (IPA Knowledge Base)
CAMK1	calcium/calmodulin dependent protein kinase I	-0.627	Inhibition
MEF2C	myocyte enhancer factor 2C	0.767	Activation
PLCE1	phospholipase C epsilon 1	1.333	Activation
PPP3CB	protein phosphatase 3 catalytic subunit beta	0.581	Activation
PPP3R1	protein phosphatase 3 regulatory subunit B, alpha	-1.267	Activation

NFAT = nuclear factor of activated T cells; IPA **=** Ingenuity Pathway Analysis.

**Table 7:** Analysis of mRNA Levels via Quantitative RT-PCR and RNA Microarray

Gene Name	MR	NC	*P* Value
PPP3CB (P)	14.45±0.35	16.02±0.19	0.007
PPP3CB (M)	1564.31±86.50	1039.52±45.45	0.017
PLCE1 (P)	15.68±0.42	17.16±0.42	0.079
PLCE1 (M)	971.23±49.64	383.49±19.73	0.017
CAMK1 (P)	18.57±0.24	17.43±0.43	0.043
CAMK1 (M)	166.52±6.75	256.12±7.70	0.017
PPP3R1 (P)	14.69±0.42	13.06±0.27	0.017
PPP3R1 (M)	253.76±29.68	595.10±97.05	0.017
MEF2C (P)	15.67±0.56	17.82±0.36	0.021
MEF2C (M)	638.07±63.74	366.23±29.81	0.017

Data are presented as mean ± SEM.

(P) = quantitative RT-PCR values (presented in △Cq units). MR (n = 14); NC (n = 6).

(M) = RNA microarray values (presented in normalized fluorescent intensity units). MR (n = 7); NC (n = 3).

Additionally, Significant Analysis of Microarrays method [7] was also applied for significant gene analysis. One thousand and eighty-three significant genes were identified with false discovery rate of 0.05, and four out of five focused genes (PLCE1, CAMK1, PPP3R1, and PPP3CB) were also identified using Significant Analysis of Microarrays method. The canonical pathway of Role of NFAT in Cardiac Hypertrophy was still significantly identified using Ingenuity Pathway Analysis with *P*-value of 0.039 and z-score of 0.83. Therefore, we focused on deciphering and discussing their regulatory roles in cardiac hypertrophy in the following sections and 5 focused NFAT associated genes (PLCE1, PPP3R1, PPP3CB, CAMK1, MEF2C) were studies for experimental validation.

### Hypertrophy of Atrial Myocytes in MR Patients Compared to Normal Subjects and Patients with Aortic Valve Disease

**Fig 4:**
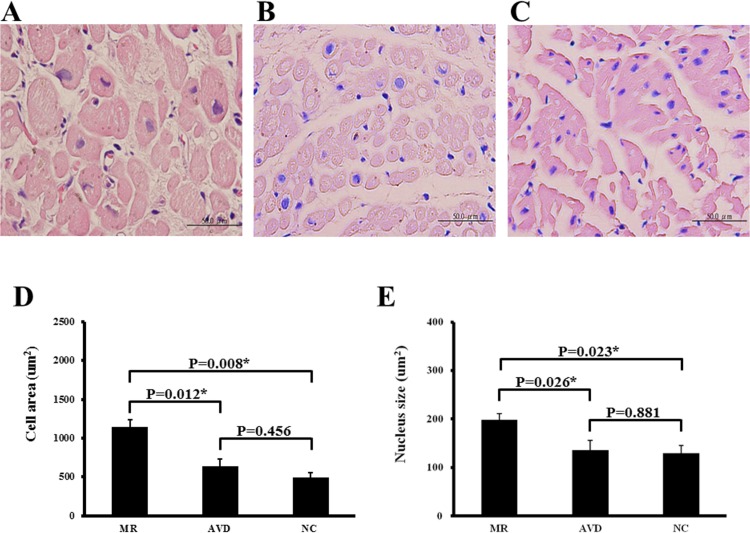
Histochemical study of hematoxylin, eosin (400 X) stained left atrial tissue sections of (**A**) mitral regurgitation (MR) patients (n = 14), (**B**) patients with aortic valve disease (AVD) (n = 5), and (**C**) normal control (NC) subjects (n = 3). The average cell surface area (**D**) and average nucleus size (**E**) per myocyte in the left atrial tissues of MR patients, patients with AVD, and normal control. *P < 0.05. Bar = 50 μm.

The average cell surface area of myocytes in the left atrial tissue of the MR patients (n = 14) significantly exceeded the average cell surface area of myocytes in the left atrial tissue of the patients with aortic valve disease (n = 5) (1145.3±97.1 vs. 637.7±95.7 μm2, *P* = 0.012) and normal control subjects (n = 3; 20-year-old Asian male and 48-year-old Asian male, purchased from Abcam, Cambridge, UK and 24-year-old Asian male, purchased from BioChain, Newark, CA, USA) (1145.3±97.1 vs. 491.7±60.7 μm2, *P* = 0.008) (Fig 4). The average nuclear size of myocytes in the left atrial tissues of the MR patients significantly exceeded the average nuclear size of myocytes in the left atrial tissue of the patients with aortic valve disease (198.8±12.0 vs. 135.7±19.8μm2, *P* = 0.026) and normal control subjects (198.8±12.0 vs. 129.9±15.1 μm2, *P* = 0.023) (Fig 4). However, the average cell surface area and nucleus size of myocytes in the left atrial tissue did not significantly differ between patients with aortic valve disease and normal subjects (*P* = 0.456 and *P* = 0.881, respectively).

### α-Sarcomeric Actin Expression in the Left Atria between MR Patients and Normal Subjects

Four normal adult left atrial tissue samples (66-year-old Caucasian female, 49-year-old Africa American male, 62-year-old Asian female and 78-year-old Caucasian female,) were purchased from BioChain, Newark, CA, USA, and these 4 normal atrial tissues were used as the normal controls for protein analysis.

The expression of α-sarcomeric actin protein (normalized against GAPDH) in the left atrial free wall was significantly up-regulated in the MR patients (n = 10) compared to normal subjects (n = 4) (1.30± 0.07 vs. 0.67± 0.13, *P* = 0.007).

### Comparison of the Gene Expression in the “Role of NFAT in Cardiac Hypertrophy” Pathway in the Left Atrium among MR Patients, Patients with Aortic Valve disease and Normal Subjects

**Fig 5:**
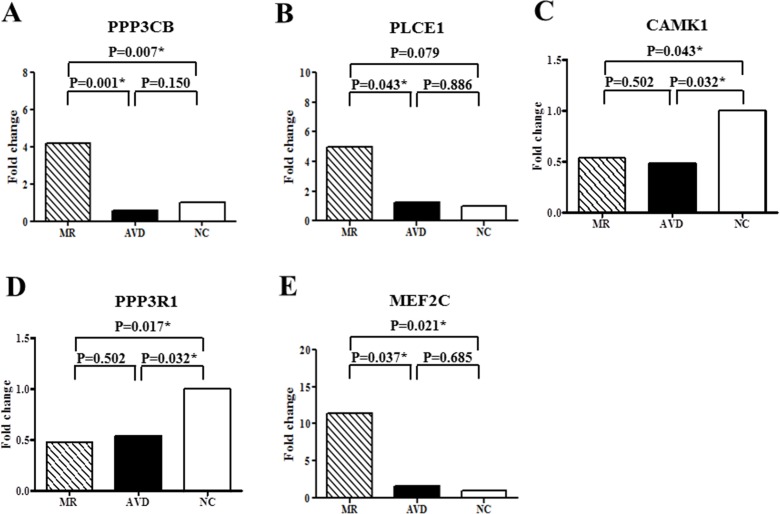
Quantitative determination of mRNAs of (**A**) protein phosphatase 3, catalytic subunit, beta isozyme (PPP3CB), (**B**) phospholipase C, epsilon 1 (PLCE1), (**C**) calcium/calmodulin-dependent protein kinase I (CAMK1), (**D**) protein phosphatase 3, regulatory subunit B, alpha (PPP3R1), and (**E**) myocyte enhancer factor 2 (MEF2C) by real-time RT-PCR in the left atrial tissues of mitral regurgitation (MR) patients, patients with aortic valve disease (AVD), and normal control (NC) subjects. *P < 0.05.

The expressions of mRNAs of PPP3CB (MR, n = 13, normal subjects, n = 6; 14.45±0.35 vs. 16.02±0.19, *P* = 0.007) and MEF2C (MR, n = 14, normal subjects, n = 5; 15.67±0.56 vs. 17.82±0.36, *P* = 0.021) in the left atrial free wall were significantly up-regulated in the MR patients compared to normal subjects (Fig 5). However, the expressions of mRNAs of CAMK1 (MR, n = 14, normal subjects, n = 6; 18.57±0.24 vs. 17.43±0.43, *P* = 0.043) and PPP3R1 (MR, n = 14, normal subjects, n = 6; 14.69±0.42 vs. 13.06±0.27, *P* = 0.017) in the left atrial free wall were significantly down-regulated in the MR patients compared to normal subjects.

The expression of mRNAs of PPP3CB (MR, n = 13, aortic valve disease, n = 6; 14.45±0.35 vs. 17.13±0.49, *P* = 0.001), MEF2C (MR, n = 14, aortic valve disease, n = 7; 15.67±0.56 vs. 17.37±0.34, *P* = 0.037) and PLCE1 (MR, n = 13, aortic valve disease, n = 7; 15.68±0.42 vs. 17.10±0.34, *P* = 0.043) in the left atrial free wall was significantly up-regulated in the MR patients compared to patients with aortic valve disease (Fig 5). However, the expression of mRNAs of CAMK1 (MR, n = 14, aortic valve disease, n = 7; 18.57±0.24 vs. 18.89±0.23, *P* = 0.502) and PPP3R1 (MR, n = 14, aortic valve disease, n = 7; 14.69±0.42 vs. 13.95±0.21, *P* = 0.502) in the left atrial free wall did not significantly differ between MR patients and patients with aortic valve disease. These findings implicated that PPP3CB, MEF2C and PLCE1 were associated with the hypertrophy of atrial myocytes in MR patients compared to patients with aortic valve disease.

The expressions of mRNAs of CAMK1 (aortic valve disease, n = 7, normal subjects, n = 6; 18.89±0.23 vs. 17.43±0.43, *P* = 0.032) and PPP3R1 (aortic valve disease, n = 7, normal subjects, n = 6; 13.95±0.21 vs. 13.06±0.27, *P* = 0.032) in the left atrial free wall were significantly down-regulated in patients with aortic valve disease compared to normal subjects (Fig 5). However, there was no significant difference in the expressions of mRNAs of PPP3CB (aortic valve disease, n = 6, normal subjects, n = 6; 17.13±0.49 vs. 16.02±0.19, *P* = 0.150), PLCE1 (aortic valve disease, n = 7, normal subjects, n = 6; 17.10±0.34 vs. 17.16±0.42, *P* = 0.886) and MEF2C (aortic valve disease, n = 7, normal subjects, n = 5; 17.37±0.34 vs. 17.82±0.36, *P* = 0.685) in the left atrial free wall between patients with aortic valve disease and normal subjects.

### Comparison of the Expressions of miR-1, miR-133 and miR-208 in the Left Atrium between MR Patients and Normal Subjects

The expression of miR-1 in the left atrial tissue was significantly down-regulated in the MR patients (n = 5) compared to normal controls (n = 5) (-2.794±0.561 vs. -5.252±0.807, *P* = 0.047). The expression of miR-133 in the left atrial tissue was significantly down-regulated in the MR patients (n = 5) compared to normal controls (n = 5) (-0.065±0.334 vs. -2.083±0.691, *P* = 0.028). The expression of miR-208 in the left atrial tissue was significantly up-regulated in the MR patients (n = 5) compared to normal controls (n = 5) (-0.995±0.415 vs. 1.460±0.918, *P* = 0.028).

### Hypertrophy of HL-1 Atrial Myocytes by Mechanical Stretching

**Fig 6:**
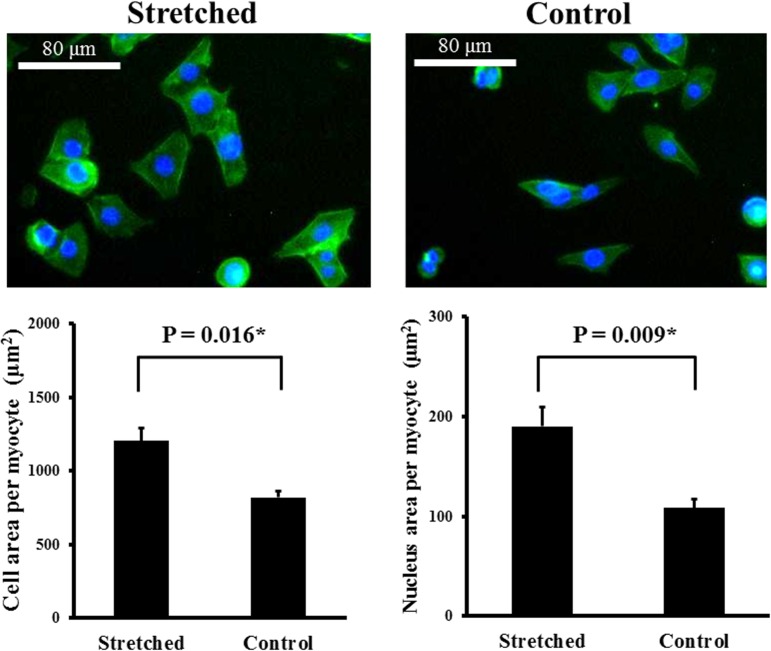
Immunofluorescence study of average cell surface area and average nucleus size of HL-1 atrial myocytes between the stretched group and non-stretched control group. Myocyte identification was performed with Phalloidin F-actin (green color). Nucleus identification was performed with Hoechst 33258 (blue color). *P < 0.05. Bar = 80 μm.

The causal relationship between atrial hypertrophy and atrial dilatation due to volume overload of MR was partly mimicked by mechanical stretching of HL-1 atrial myocytes. The average cell surface area of stretched HL-1 atrial myocytes (experiment number = 6) significantly exceeded that of non-stretched control group (experiment number = 6) (1203.6±89.0 vs. 819.4±43.1 μm2, *P* = 0.016). The average nucleus size of stretched HL-1 atrial myocytes significantly exceeded that of non-stretched control group (190.2±18.9 vs. 108.3±8.6 μm2, *P* = 0.009) (Fig 6).

bgThe gene expressions of CAMK1 (4.22±0.07 vs. 4.01±0.06, *P* = 0.037) and PPP3R1 (3.89±0.08 vs. 3.09±0.06, *P* = 0.004) (normalized against GAPDH) in the HL-1 atrial myocytes were significantly down-regulated in the stretched HL-1 atrial myocytes compared to the non-stretched control group.

## Discussion

This study identifies and reports the alteration of the RNA expression pattern, molecular mechanisms and biological processes involving in atrial myocyte hypertrophy between the left atrial myocardium of MR patients and normal subjects using high-density oligonucleotide microarrays and enrichment analysis. A total of 112 genes were identified to be differentially up-regulated and 132 genes were identified to differentially down-regulated in the left atria between MR patients and normal subjects. Notably, the expression patterns of PPP3CB, PPP3R1, PLCE1, MEF2C and CAMK1 in the “NFAT in cardiac hypertrophy” pathway played a significant role in the activation of hypertrophy of atrial myocytes in MR patients compared to normal subjects.

### Activation of “Role of NFAT in Cardiac Hypertrophy” Pathway

Calcineurin/NFAT coupling has been reported to participate in pathological, but not physiological, cardiac hypertrophy [8]. Cardiac hypertrophy is a compensatory response to pathological states and hemodynamic overload. However, pathological hypertrophy leads to atrial myocardial disarrangement and consequently, atrial enlargement that is correlated with poor prognosis in MR patients [2].

The initial phase in the development of myocardial hypertrophy involves factors, such as endothelin-1, angiotensin-II, and adrenergic agonists at the cell membrane, binding to the G-protein coupled receptors (Fig 3), and several interdependent signaling cascades that include G-proteins, GTPases such as Ras, RhoA and Rac, and kinases such as ERK/MAPK and PKC [9]. Prior study showed enhanced expression of Rho-associated kinase in the left atrial myocytes of MR patients [10]. In all of the hypertrophic pathways, NFAT plays a critical role in the development of cardiac hypertrophy. Several studies have shown the importance of Ca^2+^ sensitive signaling molecules, including calcineurins (i.e. PPP3CB, PPP3R1) and CAMK, a calcium/calmodulin-dependent protein kinase, in hypertrophic pathways [11]. Activation of protein kinase C leads to increased Ca^2+^ levels that activate calcineurins. Calcineurin activation leads to the dephosphorylation of NFATc4, allowing its nuclear localization where it cooperates with other transcription factors to participate in the cardiac hypertrophy. In this study, the expression of PPP3CB, the catalytic subunit, was significantly up-regulated in the MR patients compared to normal subjects. However, the expression of PPP3R1, the regulatory subunit B, was significantly down-regulated in the MR patients compared to normal subjects. Overexpression of PPP3R1, also known as modulatory calcineurin-interacting protein-1, has been reported to attenuate left ventricular hypertrophy after myocardial infarction [12]. As shown in Table 6, the predictive activities of the differential trend between PPP3CB and PPP3R1 to the Role of NFAT in cardiac hypertrophy pathway implicated activation of this pathway. The CAMK1 was also found to be significantly down-regulated in MR patients compared to normal subjects in this study. However, the molecular function of CAMK1 in human heart is rarely reported. Further studies are warranted to investigate the role of CAMK1 in the development of atrial hypertrophy in patients with MR.

MEF2 (myocyte enhancer factor 2), especially MEF2C, is an important transcription factor regulating the cardiac gene program during myocardial cell hypertrophy [13]. The activation of MEF2 by CAMK is mediated mainly through the phosphorylation of transcriptional repressors, the class II histone deacetylases, resulting in disrupting the association between MEF2 and histone deacetylases in the nucleus and transcriptional activation of cardiac hypertrophy [14,15]. In this study, MEF2C was found to be significantly up-regulated in MR patients compared to normal subjects.

PLCE (phospholipase C epsilon), an effector of Ras, has been shown to involve in stress-induced hypertrophy and PLCE, scaffolded to muscle-specific A kinase-anchoring protein in cardiac myocytes, responds to hypertrophic stimuli to generate diacylglycerol from phosphatidylinositol 4-phosphate in the Golgi apparatus, in close proximity to the nuclear envelope, to regulate activation of nuclear protein kinase D and hypertrophic signaling pathwa**y**s [16]. In this study, PLCE1 was found to be up-regulated in MR patients compared to patients with aortic valve disease and normal subjects.

### Hypertrophy-Related MicroRNAs

The expressions of antihypertrophic miRs, miR-1 and miR-133, in the left atrial tissue were significantly down-regulated in the MR patients compared to normal controls, while the expression of agonist of the hypertrophic response, miR-208, in the left atrial tissue were significantly up-regulated in the MR patients compared to normal controls [17].

### Hypertrophy-Related Decorin and Calponin

Decorin has been reported to promote myoblast proliferation mediated by an endoplasmic reticulum stress-related pathway [18]. Decorin was identified to be differentially down-regulated in MR patients compared to normal subjects in this study. Therefore, decorin might not be involved in the hypertrophy of atrial myocytes in MR patients, which was mainly related to the Role of NFAT in cardiac hypertrophy pathway.

Calponin has been reported to be involved in the hypertrophy of smooth muscle cells [19,20]. Calponin 2 was identified to be differentially down-regulated in MR patients compared to normal subjects in this study. Therefore, calponin 2 might not be involved in the hypertrophy of atrial myocytes in MR patients.

### Pathological Hypertrophy of Atrial Myocytes in MR

Atria, like the ventricles, can undergo hypertrophy in response to increased volume and pressure overload. In MR, the volume and pressure in the left atrium are greatly increased. The left atrium of MR patients responds by undergoing chronic dilation, which enables it to accommodate the increased volume without a large increase in pressure because of its increased compliance. However, extreme hypertrophy and dilatation is deleterious because it increases the oxygen demand of the heart and decreases mechanical efficiency. Furthermore, atrial fibrillation, an important risk factor of stroke and systemic embolization, may develop as a consequence of atrial enlargement, and vice versa [21].

### Study Limitations

There are several limitations of this study. Firstly, most of the patients with MR received renin-angiotensin system blockers. Therefore, the expression of some genes might have been modified by renin-angiotensin system blockers [22]. However, there was no significant difference in the expressions of PPP3CB (14.66±0.38 vs. 13.28±0.02, *P* = 0.167), PPP3R1 (14.29±0.41 vs. 16.16±0.91, *P* = 0.052), PLCE1 (15.95±0.46 vs. 14.22±0.02, *P* = 0.167), MEF2C (15.99±0.58 vs. 14.46±1.47, *P* = 0.392) and CAMK1 (18.49±0.29 vs. 18.86±0.38, *P* = 0.484) between MR patients with renin-angiotensin system blockers (n = 11) vs. MR patients without renin-angiotensin system blockers (n = 3). Secondly, the sample size was relatively small. However, the gene expression pattern by microarray analysis was quite consistent in the same group (Fig 1). Thirdly, the age of the normal subjects (n = 6) was younger than that of MR patients (n = 14) (49±25 vs. 58±9 years, *P* = 0.620), however the difference did not reach statistical significance. Finally, the microarrays were conducted on frozen, unsorted tissue samples. It is hence impossible to ascertain the sub-tissue or cellular origin of generated data. However, histological analysis did show atrial myocyte hypertrophy of MR patients compared to patients with aortic valve disease and normal subjects, implicating at least some involvement of cellular origin.

## Conclusions

Significant hypertrophy developed in the left atrial myocytes of MR patients compared to normal subjects and patients with aortic valve disease. The differentially expressed genes in the “Role of NFAT in cardiac hypertrophy” pathway may play a critical role in the atrial myocyte hypertrophy of MR patients and these differentially expressed genes may serve as potential targets for human MR to prevent the progression of left atrial enlargement and its related complications, such as atrial fibrillation, and heart failure.

## Accession Codes

The data discussed in this manuscript have been deposited in NCBI's Gene Expression Omnibus (GEO) and are accessible through GEO Series accession number GSE63045 (http://www.ncbi.nlm.nih.gov/geo/query/acc.cgi?acc=GSE63045).

## References

[1] Iung B, Gohlke-Bärwolf C, Tornos P, Tribouilloy C, Hall R, Butchart E, et al Working Group on Valvular Heart Disease. Recommendations on the management of the asymptomatic patient with valvular heart disease. Eur Heart J 2002;23:1253–1266. 12698958 10.1053/euhj.2002.3320

[2] Otto CM. Timing of surgery in mitral regurgitation. Heart 2003;89:100–105. 12482807 10.1136/heart.89.1.100 PMC1767516

[3] Corradi D, Callegari S, Maestri R, Ferrara D, Mangieri D, Alinovi R, et al Differential structural remodeling of the left-atrial posterior wall in patients affected by mitral regurgitation with or without persistent atrial fibrillation: a morphological and molecular study. J Cardiovasc Electrophysiol 2012;23:271–279. 10.1111/j.1540-8167.2011.02187.x 21954878

[4] Chang JP, Chen MC, Lin WY, Liu WH, Chen CJ, Chen YL, et al DNA repair in TUNEL-positive atrial cardiomyocytes of mitral and tricuspid valve diseases: potential mechanism for preserving cardiomyocytes. Int J Cardiol 2011;146:44–50. 10.1016/j.ijcard.2009.06.012 19560219

[5] Krämer A, Green J, Pollard J Jr, Tugendreich S. Causal analysis approaches in Ingenuity Pathway Analysis. Bioinformatics 2014;30:523–530. 10.1093/bioinformatics/btt703 24336805 PMC3928520

[6] Gentleman R, Carey V, Huber W and Hahne F. genefilter: genefilter: methods for filtering genes from microarray experiments. R package version 1.46.1.

[7] Tusher VG, Tibshirani R, Chu G. Significance analysis of microarrays applied to the ionizing radiation response. Proc Natl Acad Sci U S A 2001;98:5116–121. 10.1073/pnas.091062498 11309499 PMC33173

[8] Wilkins BJ, Dai YS, Bueno OF, Parsons SA, Xu J, Plank DM, et al Calcineurin/NFAT coupling participates in pathological, but not physiological, cardiac hypertrophy. Circ Res 2004;94:110–118. 10.1161/01.RES.0000109415.17511.18 14656927

[9] Kang M, Chung KY, Walker JW. G-protein coupled receptor signaling in myocardium: not for the faint of heart. Physiology 2007;22:174–184. 10.1152/physiol.00051.2006 17557938

[10] Chen HC, Chang JP, Chang TH, Lin YS, Huang YK, Pan KL, et al Enhanced expression of ROCK in left atrial myocytes of mitral regurgitation: a potential mechanism of myolysis. BMC Cardiovasc Disord 2015;15:33 10.1186/s12872-015-0038-9 25956928 PMC4429363

[11] Bueno OF, Wilkins BJ, Tymitz KM, Glascock BJ, Kimball TF, Lorenz JN, et al Impaired cardiac hypertrophic response in Calcineurin A beta -deficient mice. Proc Natl Acad Sci U S A 2002;99:4586–4591. 10.1073/pnas.072647999 11904392 PMC123691

[12] van Rooij E, Doevendans PA, Crijns HJ, Heeneman S, Lips DJ, van Bilsen M, et al MCIP1 overexpression suppresses left ventricular remodeling and sustains cardiac function after myocardial infarction. Circ Res 2004;94:e18–26. 10.1161/01.RES.0000118597.54416.00 14739160

[13] Kolodziejczyk SM, Wang L, Balazsi K, DeRepentigny Y, Kothary R, Megeney LA. MEF2 is upregulated during cardiac hypertrophy and is required for normal post-natal growth of the myocardium. Curr Biol 1999;9:1203–1206. 10.1016/S0960-9822(00)80027-5 10531040

[14] Akazawa H, Komuro I. Roles of cardiac transcription factors in cardiac hypertrophy. Circ Res 2003;92:1079–1088. 10.1161/01.RES.0000072977.86706.23 12775656

[15] Zhang CL, McKinsey TA, Olson EN. Association of class II histone deacetylases with heterochromatin protein 1: potential role for histone methylation in control of muscle differentiation. Mol Cell Biol 2002;22:7302–7312. 10.1128/MCB.22.20.7302-7312.2002 12242305 PMC139799

[16] Zhang L, Malik S, Pang J, Wang H, Park KM, Yule DI, et al Phospholipase Cε hydrolyzes perinuclear phosphatidylinositol 4-phosphate to regulate cardiac hypertrophy. Cell 2013;153:216–227. 10.1016/j.cell.2013.02.047 23540699 PMC3615249

[17] Da Costa Martins PA, De Windt LJ. MicroRNAs in control of cardiac hypertrophy. Cardiovasc Res 2012;93:563–572. 10.1093/cvr/cvs013 22266752

[18] Sun L, Lu K, Liu H, Wang H, Li X, Yang C, et al The effects of endoplasmic reticulum stress response on duck decorin stimulate myotube hypertrophy in myoblasts. Mol Cell Biochem 2013;377:151–161. 10.1007/s11010-013-1581-2 23385868

[19] di Gioia CR, van de Greef WM, Sperti G, Castoldi G, Todaro N, Ierardi C, et al Angiotensin II increases calponin expression in cultured rat vascular smooth muscle cells. Biochem Biophys Res Commun 2000;279:965–969. 10.1006/bbrc.2000.4049 11162458

[20] McWhinnie R, Pechkovsky DV, Zhou D, Lane D, Halayko AJ, Knight DA, et al Endothelin-1 induces hypertrophy and inhibits apoptosis in human airway smooth muscle cells. Am J Physiol Lung Cell Mol Physiol 2007;292:L278–286. 10.1152/ajplung.00111.2006 16920889

[21] Sanfilippo AJ, Abascal VM, Sheehan M, Oertel LB, Harrigan P, Hughes RA, et al Atrial enlargement as a consequence of atrial fibrillation. A prospective echocardiographic study. Circulation 1990;82:792–797. 2144217 10.1161/01.cir.82.3.792

[22] Chen MC, Chang JP, Chang TH, Hsu SD, Huang HD, Ho WC, et al Unraveling regulatory mechanisms of atrial remodeling of mitral regurgitation pigs by gene expression profiling analysis: role of type I angiotensin II receptor antagonist. Transl Res 2015;165:599–620. 10.1016/j.trsl.2014.11.005 25500755

